# Barriers and facilitators to implementation of menu labelling interventions from a food service industry perspective: a mixed methods systematic review

**DOI:** 10.1186/s12966-020-00948-1

**Published:** 2020-04-15

**Authors:** Claire Kerins, Sheena McHugh, Jenny McSharry, Caitlin M. Reardon, Catherine Hayes, Ivan J. Perry, Fiona Geaney, Suzanne Seery, Colette Kelly

**Affiliations:** 1 Discipline of Health Promotion, School of Health Sciences, National University of Ireland Galway, University Road, Galway, Ireland; 2 School of Public Health, University College Cork, College Road, Cork, Ireland; 3 Health Behaviour Change Research Group, School of Psychology, National University of Ireland Galway, University Road, Galway, Ireland; 4 Ann Arbor VA Center for Clinical Management Research, P.O. Box 130170, Ann Arbor, MI 48113-0170 USA; 5 Discipline of Public Health and Primary Care, Institute of Population Health, Trinity College Dublin Russell Centre, Tallaght Cross, Dublin 24, Ireland; 6 National Institute for Prevention and Cardiovascular Health, Croí Heart and Stroke Centre, Moyola Lane, Newcastle, Galway, Ireland

**Keywords:** Menu labelling, Implementation, Barriers, Facilitators, Mixed methods, Systematic review, Consolidated framework for implementation research, Best fit framework synthesis

## Abstract

**Background:**

Eating outside the home contributes to poor dietary habits worldwide and is associated with increased body fat and weight gain. Evidence shows menu labelling is effective in promoting healthier food choices; however, implementation issues have arisen. The purpose of this systematic review was to synthesise the evidence on the perceived barriers and facilitators to implementation of menu labelling interventions from the perspective of the food service industry.

**Methods:**

Peer-reviewed and grey literature were searched using databases, specialised search engines and public health organisation websites. Screening reference lists, citation chaining and contacting authors of all included studies were undertaken. Primary research studies relevant to direct supply-side stakeholders were eligible for inclusion. There were no restrictions on menu labelling scheme or format, study methods, publication year or language. At least two independent reviewers performed study selection, data extraction and quality appraisal. The results were synthesised using the ‘best fit’ framework synthesis approach, with reference to the Consolidated Framework for Implementation Research (CFIR).

**Results:**

Seventeen studies met the eligibility criteria, with the majority rated as average quality (*n* = 10). The most frequently cited barriers were coded to the CFIR constructs ‘Consumer Needs & Resources’ (e.g. lack of customer demand for/interest in menu labelling, risk of overwhelmed/confused customers) and ‘Compatibility’ with organisation work processes (e.g. lack of standardised recipes, limited space on menus). Frequently cited facilitators were coded to the CFIR constructs ‘Relative Advantage’ of menu labelling (e.g. improved business image/reputation) and ‘Consumer Needs & Resources’ (e.g. customer demand for/interest in menu labelling, providing nutrition information to customers). An adapted framework consisting of a priori and new constructs was developed, which illustrates the relationships between domains.

**Conclusion:**

This review generates an adapted CFIR framework for understanding implementation of menu labelling interventions. It highlights that implementation is influenced by multiple interdependent factors, particularly related to the external and internal context of food businesses, and features of the menu labelling intervention. The findings can be used by researchers and practitioners to develop or select strategies to address barriers that impede implementation and to leverage facilitators that assist with implementation effort.

**Trial registration:**

Systematic review registration: PROSPERO CRD42017083306.

## Background

Poor diet is a leading risk factor for chronic disease and premature death [1, 2]. Over the past decade, eating outside the home has been highlighted as one of the many factors contributing to poor dietary habits worldwide. Studies have shown that eating outside the home is associated with higher energy and fat intake, lower micronutrient intake [3–7], increased body fat and weight gain [8–11]. The average energy content of main meals served in both fast food and full service restaurants exceed public health recommendations (i.e. 600 kcal or less for main lunch/dinner meals) [12, 13]. In addition, consumers tend to underestimate the energy, fat and sodium content of meals when eating outside the home [14, 15]. Over the last decades eating outside the home has steadily increased, with fewer meals being prepared at home [5, 16, 17].

The food service industry, with responsibility for meals prepared outside the home, has an important role in promoting healthy dietary behaviours [18]. To help increase transparency in the nutritional value of meals outside the home and to assist consumers in making both informed and healthier food choices, menu labelling is recommended as part of a comprehensive approach to reduce diet-related non-communicable diseases (NCDs) [19, 20]. Menu labelling includes the provision of nutrition information on menus at the point of sale. Evidence from a recent Cochrane review suggests calorie menu labelling may reduce energy purchased per meal by an average of 8% [21]. Moreover, a recent systematic review and meta-analysis suggests menu labelling effects consumer dietary intake (i.e. 7% reduction in energy, 11% reduction in total fat and 14% increase in vegetable intake) and industry practices (i.e. reformulation of menu items to lower sodium intake by 9%) [22]. The review found no difference in effect by label type (numeric - using numerical information or interpretive - using graphics, symbols or colours) [22].

In an effort to stem the rise in obesity and other diet-related NCDs, several countries and regions around the world have introduced menu labelling on a voluntary or mandatory basis [23–27]. Similarly, a number of workplace and healthcare organisations around the world have independently developed and implemented menu labelling policies at national and local levels [28–30]. As the momentum to implement menu labelling policies has increased, issues relating to implementation have arisen. Challenges relate to delays in legislation implementation [31], lack of monitoring/enforcement [32], poor uptake by food service businesses [33, 34] and inaccurate nutritional information being presented on menus due to cited reasons such as inconsistent portioning, lack of training and difficulties in sourcing nutrition information [32, 35–37]. It is evident that the process involved in the development and implementation of labelling policies is context-specific, non-linear and shaped by many different actors and factors [38].

Most reviews have examined the effectiveness of menu labelling [21, 22], and despite evidence of implementation issues, no previous review has focused on the challenges facing the key actors responsible for adopting this intervention, the food service industry. To help systematically evaluate the determinants of implementation, the Consolidated Framework for Implementation Research (CFIR) was chosen as the a priori framework as it incorporates constructs from 19 implementation theories, frameworks and models into one single comprehensive framework [39]. Identification of barriers and facilitators (i.e. determinants) guided by an established framework will provide a foundation to select and tailor implementation strategies to improve adoption, implementation, sustainment, and scale-up of menu labelling interventions [40]. Moreover, the review will explore the relationships between determinants to assist with understanding the mechanisms underpinning the implementation of menu labelling interventions. In using the ‘best fit’ framework synthesis approach [41], an adapted CFIR framework will be developed which goes beyond listing determinants to illustrate the relationships between factors. This approach, adopted by other recently published reviews [42–45], may help advance the a priori framework towards being more testable. For example, the adapted CFIR framework can be used to generate a hypothesis, specifically in the context of menu labelling, to be tested in empirical research.

## Objectives

The review objectives were to (1) synthesise existing research on the perceived barriers and facilitators to implementation of menu labelling interventions from the perspective of the food service industry, (2) map these determinants to the CFIR constructs, (3) in instances where data does not fit the CFIR, to generate new constructs and themes, (4) identify relationships between a priori and new constructs, and (5) develop an adapted framework based on the review findings.

## Methods

The protocol, published elsewhere [46], is summarised briefly here. The review was conducted according to Preferred Reporting Items for Systematic Reviews and Meta-Analyses (PRISMA) guidelines [47] (see Additional file 1) and registered with PROSPERO, the International Prospective Register of Systematic Reviews (CRD42017083306). The review followed the steps of the ‘best fit’ framework synthesis approach [41], which starts with identification of a pre-existing framework to be used for initial coding of data which is then updated in response to the emerging synthesis, thus creating an adapted framework. This approach is described in detail in the published protocol [46].

### Conceptual framework

The CFIR is a meta-theoretical framework composed of 39 constructs under five major domains: (1) intervention characteristics, (2) outer setting, (3) inner setting, (4) individual characteristics, and (5) process [39]. The CFIR has a strong emphasis on contextual determinants, with three of the five domains (i.e. process, inner setting and outer setting) capturing contextual factors operating at multiple levels (i.e. micro, meso and macro level) [48]. The CFIR provides a common language and clear consensual definitions which allows for comparison across diverse studies [49, 50].

### Eligibility and search criteria

The criteria for study eligibility are summarised in Table 1. All primary research studies using qualitative, quantitative or mixed methods approaches were eligible for inclusion. As per the protocol [46], we had initially planned to include supply-side stakeholders (direct and indirect) with a role in implementation of menu labelling interventions; however, we decided to narrow the population of interest to direct supply-side stakeholders only (i.e. food service staff and management). These direct supply-side stakeholders are responsible for the initial decision to adopt/implement menu labelling interventions, and thus need to be targeted first to ensure adoption, implementation, sustainment, and scale-up of menu labelling interventions.

**Table 1:** Study eligibility criteria

Inclusion criteria	Exclusion criteria
Population: direct supply-side stakeholders with a role in menu labelling implementation (e.g. food service business staff and management, caterers).	Population: demand side stakeholders (e.g. consumers) and indirect supply-side stakeholders (e.g. suppliers, policy makers, guideline developers, health professionals).
Intervention: no restriction on menu labelling format (numeric or interpretive), scheme (voluntary or mandatory) or type of food service business.	Intervention: menu labelling as part of a multi-component intervention.
Type of research: all primary research studies (from grey or peer-reviewed literature) using qualitative, quantitative or mixed methods approaches.	Type of research: editorials, commentary and opinion pieces.
Language and publication year: no restrictions. Outcome: any barrier or facilitator to the implementation of menu labelling interventions.	

PubMed, EMBASE, CINAHL, PsycINFO, Web of Science and Scopus electronic databases were searched. Grey literature were sourced from: Google Scholar, OpenGrey, RIAN, EThOS, ProQuest, WorldCat, Networked Digital Library of Theses and Dissertations, Open Access Theses and Dissertations, and public health organisation websites. The search included studies up until February 2018. The search strategy is presented in Additional file 2. Screening reference lists, citation chaining and contacting authors of all included studies were undertaken.

### Study selection and appraisal

At least two independent reviewers (CK, CH, SS, CKelly, FG) conducted the study selection process (i.e. title and abstract screening followed by full text articles). The quality of the included studies were assessed by two independent reviewers (CK, JMS) using the Mixed Methods Appraisal Tool (MMAT) [51]. Reviewers resolved discrepancies through discussion and consensus. No studies were excluded based on quality scores; the quality cut-off points outlined in Appendix 6 were used as part of the sensitivity analysis to explore whether studies with different quality scores affected the presence of any constructs or domains in the adapted framework [52].

### Data extraction and synthesis

**Fig. 1:**
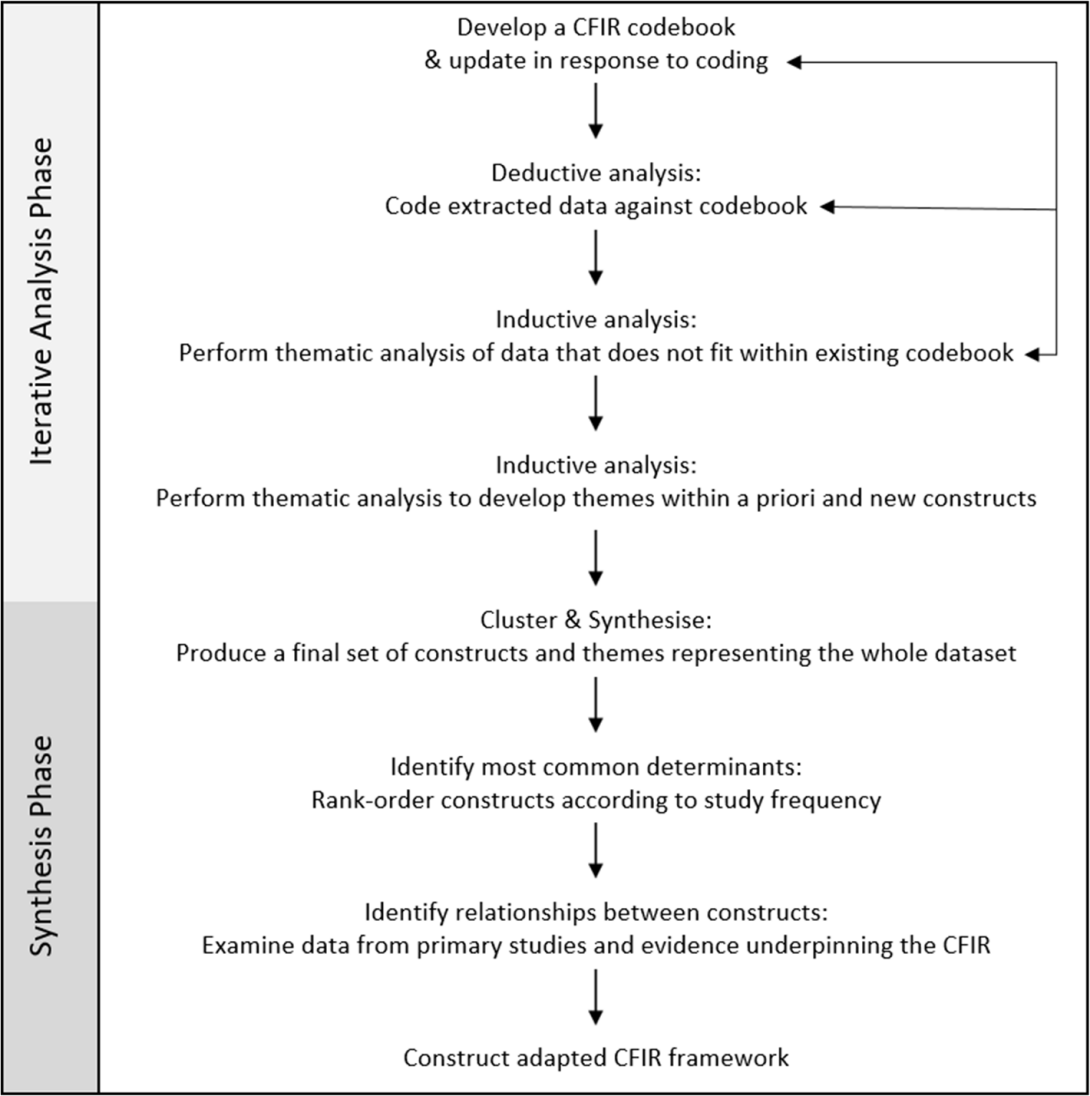
Flow diagram of data analysis and synthesis process

Two independent reviewers (CK, JMS) extracted data from all included studies (see Additional file 3 for data extraction form). Upon reaching consensus on data extracted, the synthesis was initiated (Fig. 1). Data synthesis was led by one reviewer (CK) using a mix of deductive and inductive analysis techniques, with regular consultation with review team members (SMH, CMR, CKelly) through a consensus decision making process [53, 54]. As the intervention (i.e. menu labelling) was predominantly targeted at settings outside of healthcare, the first step involved developing a codebook in which the CFIR constructs were operationalised and adapted. Deductive coding using the CFIR codebook was an iterative process, which required familiarisation with the data. Any resulting modifications to construct definitions were discussed with review team members until consensus was reached (see Additional file 4 for final codebook). Secondly, inductive coding was used to develop themes within broadly-defined CFIR constructs to capture the detail of the barrier or enabler in the context of menu labelling. Similarly, in instances where data did not fit existing CFIR constructs, thematic analysis was performed [55]. Findings were clustered and synthesised into a final set of constructs and themes representing the whole dataset.

A member of the analysis team (CMR) is from the CFIR development group and provided expert input on CFIR construct definitions during the deductive coding. Other quality checks included double-coding a sub-set of four papers (CK, CKelly), a review of constructs and supporting data following the deductive coding (CK, CMR, SMH) and a review of data that did not fit existing CFIR constructs and new constructs generated following the inductive coding (CK, CKelly, CMR, SMH). No estimates of inter-rater reliability were calculated.

Barriers and facilitators (coded to constructs) were rank-ordered according to the frequency of studies that reported them to identify the most common determinants to menu labelling implementation. In addition, review team members (CK, CMR, SMH) examined the relationships between constructs based on data from the primary research studies. These relationships were based on the results of the primary studies, i.e., the authors of the primary studies reported relationships based on their analysis of the raw data. All relationships were captured, regardless of study type, and given equal weight. In addition, a review of theories, frameworks and models underpinning the CFIR was undertaken to examine relationships between constructs/domains. The results of the coding and analysis of relationships informed the construction of an adapted CFIR framework of the implementation determinants for menu labelling interventions. The depiction of the adapted framework (as a figure) was guided by input from review team members (CK, SMH, CH, CKelly) and reviewed by all members of the team until consensus was reached.

### Testing the synthesis

Following the construction of an adapted CFIR framework, one reviewer (CK) assessed the potential for bias by examining differences between the a priori and adapted framework, and seeking evidence of negative cases, and conducted a sensitivity analysis to determine if the synthesis was sensitive to study methodology, quality and location [41].

## Results

### Study selection

**Fig. 2:**
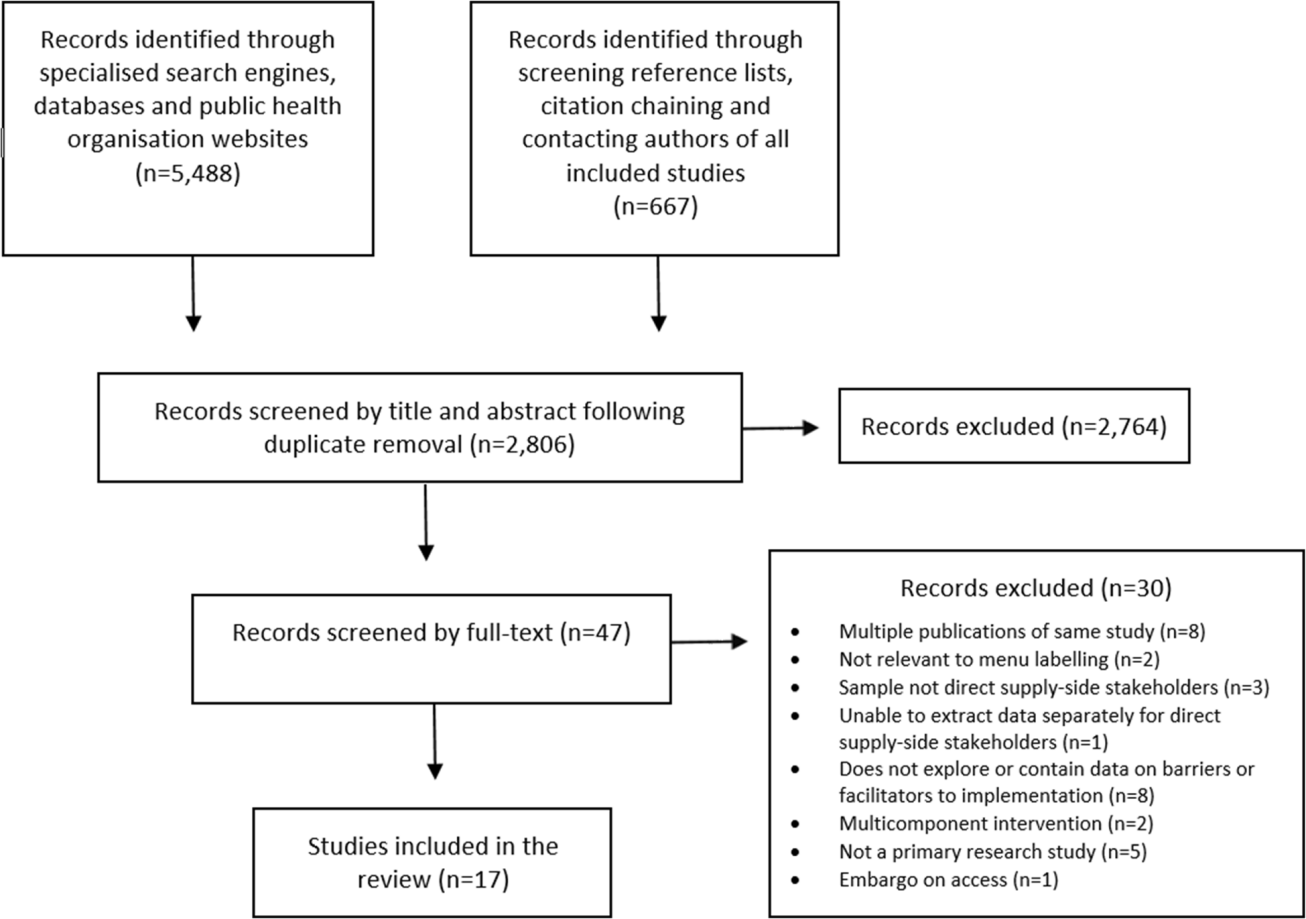
PRISMA flow diagram of study selection process

The search yielded a total of 2806 records, after duplicates were removed. Following title and abstract review, 47 records (representing 39 distinct studies) were included in the full-text review. In instances where two or more records represented the same study, the record with the most data reported was used; except in the case of two records (peer-reviewed article in English and dissertation in Portuguese) representing one study, the less comprehensive dataset available in English was selected to avoid issues with language translation. Following full-text review, 17 studies were included (Fig. 2).

### Study characteristics and quality appraisal

Study characteristics are provided in Additional file 5. Included studies were largely based in developed countries (*n* = 15). Studies used mostly quantitative data collection methods (*n* = 8), followed by qualitative (*n* = 7) and mixed methods (*n* = 2). Only two studies used an explicit theory and in both instances, the Diffusion of Innovation Theory [56, 57]. The study setting ranged from small independently owned restaurants to large foodservice corporations including chain and franchise restaurants, as well as large catering companies. Menu labelling schemes were predominately voluntary (*n* = 16), with one study including both voluntary and mandatory schemes [58]. Most studies assessed both pre- and post-implementation of menu labelling (*n* = 9), while the remaining studies examined pre-implementation (*n* = 3) and post-implementation (*n* = 5). In terms of study quality, the majority of studies were rated as average quality (*n* = 10). Additional file 6 presents a detailed breakdown of the quality assessment for each study.

### Barriers and facilitators

A summary of perceived barriers and facilitators coded to the CFIR constructs, including constructs with no supporting data, are presented in Table 2. New constructs generated inductively from the data, that did not align with existing CFIR constructs, are presented in Table 3 (see definitions and eligibility criteria in Additional file 4). While some a priori and new constructs manifested as either barriers or facilitators to implementation, others acted simultaneously as barriers and facilitators (e.g. increased consumer demand for menu labelling or the lack thereof). Additional file 7 provides more detailed results, including themes with references under each construct and illustrative quotes. A narrative summary of frequently cited perceived barriers and facilitators (i.e. those discussed in 10 or more of the included studies) is presented below.

**Table 2:** Summary of perceived barriers and facilitators to implementation of menu labelling coded to the CFIR

CFIR domain	CFIR construct	CFIR sub-construct	Studies that identified facilitators	Studies that identified barriers
Intervention Characteristics	Intervention source		[59–61]	No data
	Evidence strength and quality		[62, 63]	[62, 64, 65]
	Relative advantage		[56–70]	[59, 61–65, 67–71]
	Adaptability		No data	No data
	Trialability		No data	No data
	Complexity		No data	[59–63, 65, 70, 71]
	Design quality and packaging		[63, 67]	[59, 61, 65, 68]
	Cost		[61]	[58, 59, 61–66, 68, 69, 71, 72]
Outer Setting	Consumer needs and resources		[57–65, 67, 68, 72]	[57–68, 70, 71]
	Cosmopolitanism		[59, 61]	No data
	Peer pressure		[57–61, 63, 64, 67]	[61]
	External policy and incentives		[56, 58–63, 67, 68]	[58, 59, 61–66, 68–71]
Inner Setting	Structural characteristics		[57, 61, 63–65]	[60–63, 65, 66]
	Networks and communications		[70]	No data
	Culture		No data	No data
	Implementation climate			
		Tension for change	No data	[61, 62, 65, 68, 71]
		Compatibility	[58–64, 69, 71]	[58–66, 69–71]
		Relative priority	No data	[60–64, 71]
		Incentives and rewards	No data	[62]
		Goals and feedback	[58, 61, 69]	[59, 61, 62, 64, 67–71]
		Learning climate	No data	No data
	Readiness for implementation			
		Leadership support	[61, 62]	No data
		Available resources	[62, 63, 65]	[58–63, 65, 68, 69, 71]
		Access to knowledge and information	[57, 59–63, 65–67, 70]	[58–63, 66, 69, 71, 72]
Characteristics of Individuals	Knowledge and beliefs		[61, 63, 67]	[60, 62, 65, 69, 71, 72]
	Self-efficacy		No data	No data
	Individual stage of change		No data	No data
	Individual identification with food business		No data	No data
	Other personal attributes		[71]	[60, 71]
Process	Planning		No data	No data
	Engaging		[63]	No data
		Opinion leaders	[59]	No data
		Formally appointed internal implementation leaders	No data	No data
		Champions	No data	No data
		External change agents	[59–63, 65, 67]	[60, 61, 63]
	Executing		No data	[58, 61, 62]
	Reflecting and evaluating		[56]	No data

**Table 3:** Summary of new constructs generated inductively from the data

CFIR domain	New construct	Studies that identified facilitators	Studies that identified barriers
Outer Setting	Media & societal pressure	[58, 67]	No data
	Economic climate	No data	[62, 63, 65]
	Educational system	[61, 63]	No data
Process	Engaging: internal key stakeholders	[59]	[59, 61–63, 69, 71]
	Engaging: external key stakeholders	[63, 65]	[59–63, 69, 71]
	Adapting the organisation	[61, 63, 70]	No data
	Adapting the intervention	[59, 61, 62, 65, 67, 68, 70, 71]	No data
	Trialing	[63]	No data
	Scaling up	[59, 63]	No data

### Barriers

The most commonly reported barriers to implementation were coded to the CFIR constructs “Implementation Climate” (14/17) and “Consumer Needs and Resources” (13/17). More specifically, within the construct “Implementation Climate”, frequently reported barriers were coded to the sub-construct “Compatibility” (12/17). Barriers relating to “Compatibility” (i.e. compatibility with organisation work processes) included lack of standardised recipes/menus, limited space on menus, frequent menu changes/variations, too many products/menu items, no sense of responsibility, lack of alignment with food served/aesthetics, fast-paced environment and lack of mass-produced food. Barriers relating to “Consumer Needs and Resources” (i.e. consumer needs and preferences) included lack of customer demand for/interest in menu labelling, risk of overwhelmed/confused customers and interference with customer dining experience.

Other frequently reported barriers were coded to the CFIR constructs “Cost” (12/17), “Relative Advantage” (11/17) and “External Policies and Incentives” (12/17). Barriers relating to “Cost” (i.e. cost of the intervention) included the general cost of menu labelling, nutritional analysis, changing menu/displays, printing nutrition information, hiring consultants/appointing advisors, purchasing nutrition analysis software, staff time in implementation and finally, the cost of training staff. Barriers relating to “Relative Advantage” (i.e. (dis) advantage of implementing intervention compared to alternative) included reduced sales/profitability, loss of flexibility/creativity, customer loss, negative impact on image/brand and lack of economic return on time investment. Barriers relating to “External Policies and Incentives” (i.e. external policies to spread intervention) included absence of legislation, lack of monitoring and enforcement, lack of guidelines, fears of liability due to inaccurate information and perceived excessive bureaucratic burdens on businesses.

Frequently reported barriers were also coded to the CFIR construct “Readiness for Implementation” (12/17). Under “Readiness for Implementation”, commonly reported barriers were coded to the sub-constructs “Available Resources” (10/17) and “Access to Knowledge and Information” (10/17). Barriers relating to “Available Resources” (i.e. available resources within the organisation) included lack of time, lack of money, limited staff and short-term building lease. Barriers relating to “Access to Knowledge and Information” (i.e. access to knowledge and information about the intervention) within the implementing food service business included lack of nutrition expertise in the organisation, challenges in acquiring nutrition information, lack of reliable nutrition information, lack of training and support, lack of nutrition analysis software and difficulties with business systems. There were also barriers to ‘accessing knowledge and information’ from agencies external to the food service businesses, which included difficulty in obtaining information from suppliers/health agencies, discrepancy in nutrition information obtained from suppliers and excessive information provided by health agencies.

### Facilitators

The most commonly reported facilitators were coded to the CFIR constructs “Relative Advantage” of menu labelling (15/17) and “Consumer Needs and Resources” (12/17). Facilitators relating to “Relative Advantage” included perceived benefits of menu labelling for participating businesses, improved business image/reputation, attracting/retaining customers, increased sales/profitability, cost saving, increased customer trust/confidence, opportunity for better customer service, opportunity for creativity/learning, menu labelling as a marketing tool, building relationships with health authorities. Facilitators relating to “Consumer Needs and Resources” included consumer demand for/interest in menu labelling, providing nutrition information to customers, enabling informed food choices, providing a consistent menu labelling scheme for customers and improving customer health.

Frequently cited facilitators were also coded to the CFIR construct “Readiness for Implementation” (10/17). More specifically, within the construct “Readiness for Implementation”, frequently reported facilitators were coded to the sub-construct “Access to Knowledge and Information” (10/17). Facilitators relating to “Access to Knowledge and Information” within the implementing food service business included staff training and support, access to information, access to (user-friendly) nutrition analysis software and access to an in-house technician. There were also facilitators to ‘accessing knowledge and information’ from agencies external to the food service businesses, which included access to knowledge and information from designers/professionals/health agencies/suppliers, access to user-friendly (approved) nutrition analysis software from health agencies and access to training from professionals/health agencies.

### Construct relationships

Relationships between constructs, within and/or across domains, were evident in 14 out of 17 studies; where one or more constructs facilitated and/or hindered one or more additional constructs. For example, being part of a large franchise (i.e. “Structural Characteristics”) facilitated access to standardised recipes and information (i.e. “Compatibility” and “Access to Knowledge and Information”) [63] . In 5 out of 17 studies, there was a relationship between the barriers encountered and the way the intervention was tailored or may be tailored for future scale. For example, in response to liability concerns due to inaccurate nutritional information (i.e. “External Policy and Incentives”), businesses included a caveat on their menus stating reasons for possible variation from stated values (i.e. “Adapting the Intervention”) [59]. Another example includes a perceived lack of consumer knowledge and non-user friendly menu labels (i.e. “Consumer Needs and Resources” and “Design Quality and Packaging) which led businesses to recommend a consumer education campaign to be rolled out alongside future menu labelling interventions (i.e. “Adapting the Intervention”). For more detailed information on construct relationships, including illustrative quotes and reference studies, see Additional file 8.

### Framework revision

**Fig. 3:**
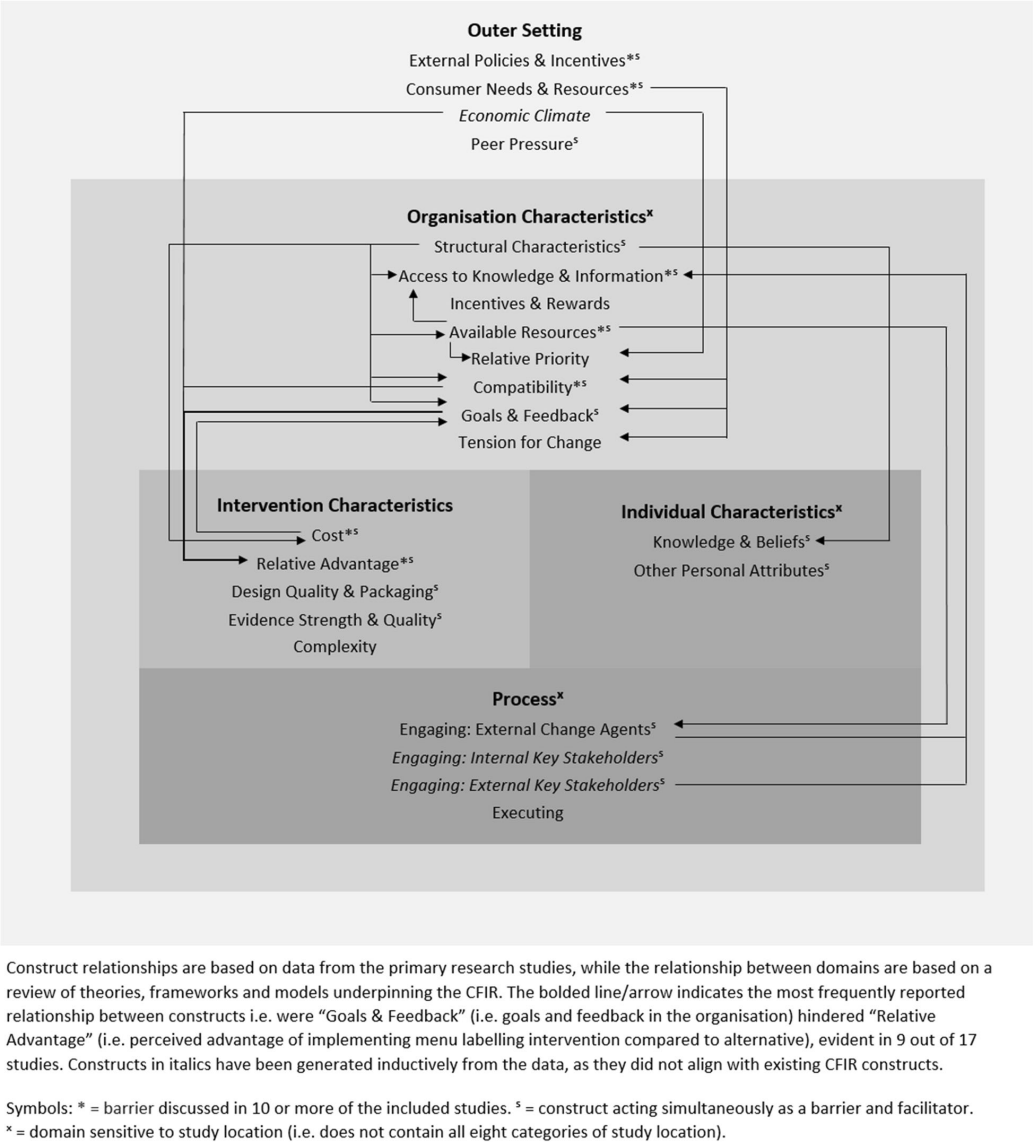
Adapted ‘Consolidated Framework for Implementation Research’ of the barriers to implementing menu labelling interventions across multiple levels. Outer Setting = external environment to food service businesses; Organisation Characteristics = internal environment of food service businesses; Intervention Characteristics = features of menu labelling intervention; Individual Characteristics = characteristics of individuals within food service businesses; Process = process of implementing menu labelling intervention

**Fig. 4:**
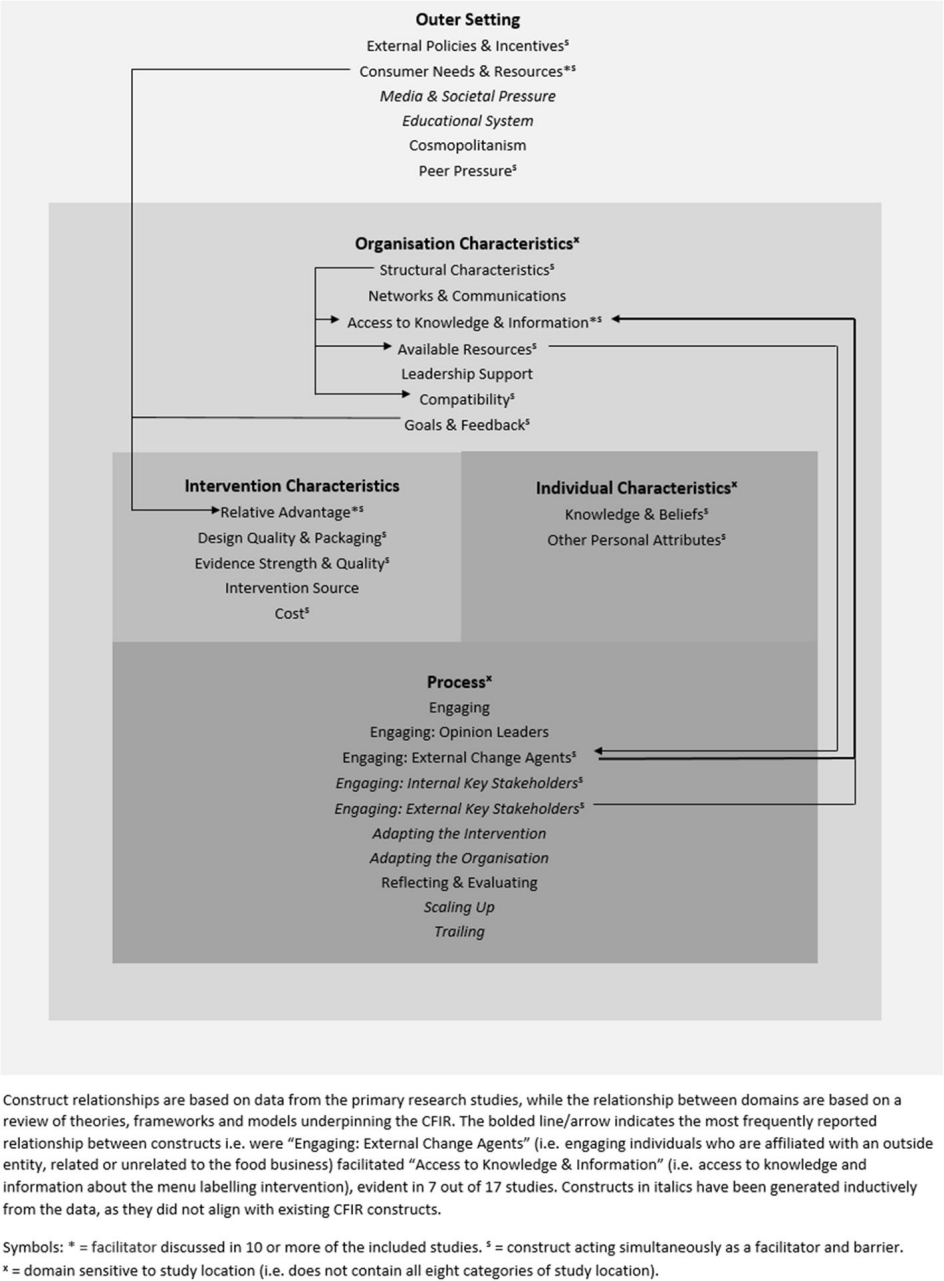
Adapted ‘Consolidated Framework for Implementation Research’ of the facilitators to implementing menu labelling interventions across multiple levels. Outer Setting = external environment to food service businesses; Organisation Characteristics = internal environment of food service businesses; Intervention Characteristics = features of menu labelling intervention; Individual Characteristics = characteristics of individuals within food service businesses; Process = process of implementing menu labelling intervention

The adapted framework maintained many of the elements of the CFIR but a number of (sub) constructs with no supporting data were removed and newly developed constructs incorporated. For clarity and ease of understanding, the barriers and facilitators to implementation of menu labelling interventions are illustrated separately in Figs. 3 and 4, respectively. The adapted framework, consisting of the five original CFIR domains, also illustrates the relationship between these domains; three domains (i.e. “Intervention Characteristics”, “Individual Characteristics” and “Process”) are nested within the “Organisation Characteristics” domain, while the “Organisation Characteristics” is nested within the “Outer Setting” domain. This depiction reflects the influence of the external context (e.g. legislation, economy, consumers) on the internal setting of food service businesses (e.g. ways of working, perception of importance of implementing menu labelling) and subsequently, their contribution to shaping positive or negative perceptions of the menu labelling intervention itself (e.g. perceived benefits of menu labelling compared to an alternative), the individuals involved in implementation (e.g. their knowledge and beliefs about menu labelling) and the process involved in implementation (e.g. refining workflows to accommodate menu labelling, engaging relevant stakeholders).

### Sensitivity analysis

Sensitivity analysis showed the adapted framework was sensitive to a number of factors including study methodology, quality and study location. While all overarching domains were not sensitive to study methodology or quality, the following domains were sensitive to study location: “Inner Setting”, “Characteristics of Individuals” and “Process” (illustrated in Figs. 3 and 4). None of these domains contained all eight categories of study location. Constructs common to all study locations included: “Relative Advantage”, “Consumer Needs & Resources” and “External Policy & Incentives”. For more detailed results of the sensitivity analysis, see Additional file 9. No constructs or domains were removed from the adapted framework based on the sensitivity analysis undertaken.

## Discussion

The review identified a range of perceived barriers and facilitators to implementation of menu labelling interventions, using an existing conceptual implementation framework (i.e. the CFIR) [39]. Factors influencing implementation were predominantly related to key characteristics of the menu labelling intervention as well as factors operating within the inner setting of food service businesses and external context of food service businesses. Multiple cases of dissonance (i.e. contradictory views) were also evident in the review findings, with many a priori and new constructs acting simultaneously as barriers and facilitators. Furthermore, interrelationships between constructs within and across domains were also evident, highlighting the complex and dynamic influences at play during implementation. A key output of the review was an adapted framework which was constructed to illustrate how factors interact to influence implementation effectiveness of menu labelling interventions.

The structure of the adapted framework (Figs. 3 & 4) somewhat reflects some of the theories/models underpinning the CFIR [73–76] as well as key organisational theories [77]. These theories/models highlight the influence of the complex interaction between organisations and their environment. In particular, organisational theories highlight that optimal conditions in the internal setting of organisations can be undermined by changes in the organisations’ external environment [78–82]. Moreover, policy implementation research has emphasised the inherent interdependency between factors as well as the crucial importance of context [83]. In the adapted framework, the food service business is embedded in their external environment to reflect the interaction between both. Nested within the food service business includes characteristics of individuals involved, features of the menu labelling intervention and the process of implementation. This structure confirms prior research and theory that implementation success is influenced by multi-level contextual factors (i.e. individual, organisational and system-level factors) [48, 83].

The findings of this review echo propositions of organisational theories which suggest implementation is more likely to occur if innovations promote organisational survival [77, 84]. For example, organisations will implement innovations to comply with accrediting bodies [85], to respond to changing patient needs [86] and to offer a valuable service to patients [87]. Similarly, in an effort to promote survival, organisations will minimise costs; adhere to norms, values and expectations adopted by institutions in their environment; develop characteristics that differentiate them from competitors; and acquire/maintain resources and autonomy [84]. In this review, frequently cited factors influencing menu labelling implementation were coded to the CFIR (sub)constructs: “External Policy and Incentives”, “Consumer Needs and Resources”, “Peer Pressure”, “Compatibility”, “Available Resources”, “Relative Advantage” and “Cost”.

Many theories underpinning the CFIR have focused on implementation of clinical innovations in healthcare and mental health settings; thus, the application of the CFIR to a public health intervention (i.e. menu labelling) in the food service setting may explain the absence of a priori constructs in the data and the addition of new constructs. In particular, constructs under the “Characteristics of Individuals” domain were infrequently coded. This may be due to CFIR’s predominate focus on determinants of implementation at organisation and system levels rather than individual level. Thus, the CFIR may not provide sufficient individual-level constructs and operationalisation of these to assist with coding. Newly developed constructs that were frequently coded (i.e. “Engaging: internal key stakeholders” and “Engaging: external key stakeholders”) are in line with proposed changes for the second version of the CFIR, which will include the sub-construct “Engaging: key stakeholders” under the “Process” domain [88].

### Implications and recommendations for practice, policy, and research

Our identification of the influential factors in the implementation of menu labelling can help guide policy/decision makers in selecting and tailoring implementation strategies [40, 89]. The Expert Recommendations for Implementing Change (ERIC) project provides a taxonomy of discrete implementation strategies that can be used in isolation or in combination to target factors influencing implementation at different levels [90]. More recently, the CFIR-ERIC Implementation Strategy Matching Tool has been developed to match ERIC implementation strategies to address CFIR-based barriers [91]. Despite being a useful starting point, this tool should be used with caution due to the lack of consensus on which ERIC strategies best address CFIR-based contextual barriers [91]. Nevertheless, the tool can be used alongside implementation mapping [92], a systematic and rigorous approach based on intervention mapping [93, 94], to help further develop or identify strategies to address contextual determinants of menu labelling implementation.

One strategy recommended by food businesses is the development of a convincing business case for menu labelling, framing the ‘relative advantage’ of menu labelling in order to attract food businesses [63]. Studies suggest food businesses are willing to overcome perceived barriers (e.g. cost, standardising recipes) in order to obtain the perceived benefits (e.g. improved business image, attracting customers) [60, 61, 63]. Moreover, there are potential cost-saving implications from menu labelling (via removing ingredients and standardising recipes/serving sizes) that may attract food businesses [60, 63]. While research shows menu labelling effects consumer behaviour [21, 22], the review findings suggest food businesses rely more on evidence from consumers/business activity than scientific research. Despite their being evidence of public support for menu labelling [64, 65, 95], further efforts are required to engage consumers in order to increase uptake and demand (e.g. through consumer education mass media campaigns) [96, 97].

In the review, a commonly reported facilitator and recommendation was that of adaptation. Food businesses adapted or recommended adapting the menu labelling intervention to help overcome perceived barriers (e.g. lack of consumer understanding, limited space on menus). While adaptation can be viewed as a key step in the implementation process [98, 99], the risk of compromising fidelity to core intervention components and potentially reducing the effects needs to be considered [100]. Few of the included studies adequately reported on the intervention components of their menu labelling scheme; thus future studies should report on same using the TIDieR-PHP checklist [101]. Moreover, research should investigate how menu labelling interventions can be effectively adapted to different contexts (e.g. large chain versus small independently-owned restaurants) without jeopardising core intervention components and effect size.

Another prominent barrier and facilitator was that of policy/legislation relating to menu labelling. Food businesses reported adopting menu labelling in response to mandatory schemes, but not in the absence of same. This finding is in line with key propositions of institutional theory, which suggest organisations will implement an innovation in response to coercion or strong pressure to comply with rules, mandates or regulations [102]. While it is recognised that regulation is required to advance public health [19, 38, 103], the risk of tokenism, that is the superficial implementation of menu labelling, in response to regulation needs to be averted [102]. In the absence of monitoring and enforcement, food businesses may present inaccurate nutrition information on menus [32]. This highlights the need for effective mechanisms to be put in place to ensure rigorous enforcement alongside regulation [32, 104].

Future studies should determine the phase of implementation relevant to different factors. Few implementation models/frameworks, including the CFIR [39], recognise that different factors may influence implementation at different points in the implementation process [105]. Such research may help identify factors which manifest or present more prominently at different stages of implementation allowing stakeholders to anticipate and address factors in sequence or in tandem for effective implementation.

Future research should investigate the relationships between the determinants. To date, many implementation studies have assessed determinants individually, assuming a linear relationship between the determinants and the outcomes [106]. However, there may be a synergistic negative effect between two seemingly minor barriers which constitute an important obstacle to effective implementation when they interact [106]. Moreover, the nature of the relationship should be examined in future studies. For example, interrelationships between factors may break down into more specific types of relationships (e.g. leverages/mitigates, engages/disengages). Future research should also assess the relative importance of determinants and relationships. Furthermore, this first review of the evidence is a starting point from which researchers can test relationships and refine the proposed adapted framework.

Finally, this review adopted a bottom-up policy implementation perspective by focusing on the views of direct supply-side stakeholders (i.e. food service staff and management), a decision based on this group being key stakeholders in the decision to adopt/implement menu labelling [83]. According to public health advocates, industry actors should not be involved in nutrition policy development due to concerns over conflict of interest; however, they should be involved during implementation [38, 97, 103, 104]. Interestingly, findings from this review suggest intervention source was not a perceived barrier to implementation, but a facilitator in a limited number of studies. As implementation of food policies (such as menu labelling) are influenced by multiple actors and factors, future research should consider the perspective of indirect supply-side stakeholders (i.e. policy-makers, researchers, public health professionals, food suppliers) as well as demand-side stakeholders (i.e. consumers).

### Limitations

Despite using comprehensive and rigorous methods in this review, there were limitations that must be acknowledged. Firstly, most of the included studies were cross-sectional; therefore, we cannot state with certainty the factors that represent the most important influence. Moreover, the relative importance of different factors and relationships is likely to be context dependent. The review found heterogeneity in terms of study setting, ranging from small independently owned restaurants to large foodservice corporations including chain and franchise restaurants, as well as large catering companies. The review also showed many a priori and new constructs acted simultaneously as barriers and facilitators, which highlights heterogeneity in the findings. Secondly, determinants of implementation were often assessed individually in the included studies; thus, (implicitly) assuming a linear relationship between the determinants and the outcomes. Therefore, the full extent of relationships between individual barriers and facilitators may not be captured in the primary studies. Thirdly, the majority of studies used questionnaires/surveys which are subject to bias due to the researcher’s selection of determinants and so studies may not have captured all relevant barriers and facilitators. Fourthly, included studies often failed to distinguish between actual barriers and facilitators experienced and those perceived to exist. The review findings may place equal importance on all determinants, although perceived importance of particular factors may not correspond with actual importance. Finally, despite undertaking and reporting on quality appraisal and a sensitivity analysis, all studies were synthesised equally in order to provide a literature summary. Therefore, evidence from studies may have been given undue weight and others underemphasised.

## Conclusion

This systematic review generates an adapted CFIR framework for understanding implementation of menu labelling interventions. The adapted framework highlights that implementation of menu labelling is influenced by multiple interdependent factors, particularly related to the external context of food businesses (e.g. consumers, legislation), internal setting of food businesses (e.g. compatibility, available information and resources) and features of the menu labelling intervention (e.g. perceived benefits, cost). The findings can be used by researchers and practitioners to develop, select, tailor and test strategies to address barriers that impede implementation and to leverage facilitators that assist with implementation effort. Future research should build on this work in order to assess the relative importance of determinants and relationships.

## Supplementary information

**Additional file 1.** PRISMA (Preferred Reporting Items for Systematic Reviews and Meta-Analyses) 2009 checklist. This file provides a completed PRISMA 2009 checklist.

**Additional file 2.** Review search strategy. This file provides the search strategy used in this review.

**Additional file 3.** Standardised data extraction form. This file provides the data extraction form used in this review.

**Additional file 4.** Final codebook and coding assumptions. This file provides the final codebook and coding assumptions used in this review.

**Additional file 5.** Characteristics of included studies. This file provides information on the characteristics of included studies in this review.

**Additional file 6.** Quality assessment of each study using MMAT criteria. This file provides a detailed breakdown of the quality assessment for each study.

**Additional file 7.** List of constructs and sample quotes following the deductive and inductive coding. This file provides results from the deductive and inductive coding, including themes under each construct and illustrative quotes.

**Additional file 8.** Construct relationships. This file provides information on construct relationships and recommendations, including illustrative quotes.

**Additional file 9.** Sensitivity analysis results for each construct. This file provides results of the sensitivity analysis.

## Data Availability

Not applicable

## Abbreviations

CFIR: Consolidated Framework for Implementation Research
ERIC: Expert Recommendations for Implementing Change
MMAT: Mixed Methods Appraisal Tool
NCDs: Non-communicable diseases
PRISMA: Preferred Reporting Items for Systematic Reviews and Meta-Analyses
