# Supplementary material for: Barriers and facilitators to implementation of menu labelling interventions from a food service industry perspective: a mixed methods systematic review
Source: Int J Behav Nutr Phys Act. 2020 Apr 15;17:48. doi: 10.1186/s12966-020-00948-1 (PMC7161210; doi:10.1186/s12966-020-00948-1)
Supplement: Supplementary file 2 — Additional file 2. Review search strategy. This file provides the search strategy used in this review. [file 12966_2020_948_MOESM2_ESM.docx]

**Additional file 2** Review search strategy

1. **PubMed**

Search string: (restaurant* [tiab] OR cafeteria* [tiab] OR canteen* [tiab] OR fast food [tiab] OR vending machine* [tiab] OR menu* [tiab] OR food service [mh] OR food service* [tiab]) AND (food labeling [mh] OR label* [tiab] OR post* [tiab]) AND (calorie* [tiab] OR kilojoule* [tiab] OR energy [tiab] OR nutri* [tiab])

1. **Scopus**

Search string: (TITLE-ABS-KEY(restaurant*) OR TITLE-ABS-KEY(cafeteria*) OR TITLE-ABS-KEY(canteen*) OR TITLE-ABS-KEY("fast food") OR TITLE-ABS-KEY("vending machine*") OR TITLE-ABS-KEY(menu*) OR TITLE-ABS-KEY("food service*")) AND (TITLE-ABS-KEY(label*) OR TITLE-ABS-KEY(post) OR TITLE-ABS-KEY(posts) OR TITLE-ABS-KEY(posting) OR TITLE-ABS-KEY(postings)) AND (TITLE-ABS-KEY(calorie*) OR TITLE-ABS-KEY(kilojoule*) OR TITLE-ABS-KEY(energy) OR TITLE-ABS-KEY(nutri*))

1. **CINAHL Complete**

Search string: (AB restaurant* OR AB cafeteria* OR AB canteen* OR AB “fast food*” OR AB “vending machine*” OR AB menu* OR AB “food service*” OR MH “food services+”) AND (MH “food labeling” OR AB label* OR AB post OR AB posts OR AB posting OR AB postings) AND (AB calorie* OR AB kilojoule* OR AB energy OR AB nutri*)

1. **EMBASE**

Search string: ('catering service'/syn OR restaurant*:ab,ti OR cafeteria*:ab,ti OR canteen*:ab,ti OR 'fast food':ab,ti OR 'vending machine*':ab,ti OR menu*:ab,ti OR 'food service*':ab,ti) AND ('food labeling'/syn OR label*:ab,ti OR post:ab,ti OR posts:ab,ti OR posting:ab,ti OR postings:ab,ti) AND (calorie/syn OR calorie*:ab,ti OR kilojoule*:ab,ti OR energy:ab,ti OR ‘nutritional value’/syn OR nutri*:ab,ti)

1. **PsycINFO**

Search string: (restaurant*.tw OR cafeteria*.tw OR canteen*.tw OR fast food.tw OR fast food.sh OR vending machine*.tw OR menu*.tw OR food service*.tw) AND (labeling.sh OR label*.tw OR post.tw OR posts.tw OR posting.tw OR postings.tw) AND (calories.sh OR calorie*.tw OR kilojoule*.tw OR energy.tw OR nutri*.tw)

1. **Web of Science Core Collection**

Search string: (TS=(restaurant$) OR TS=(cafeteria$) OR TS=(canteen$) OR TS=("fast food") OR TS=("vending machine$") OR TS=(menu$) OR TS=("food service$")) AND (TS=(label*) OR TS=(post$) OR TS=(posting$)) AND (TS=(calorie$) OR TS=(kilojoule$) OR TS=(energy) OR TS=(nutri*))

1. **Google Scholar**

First 200 results retrieved by the following search:

With all of the words: implement, stakeholder; with at least one of the words: "menu label" OR "menu labels" OR "menu labeled" OR "menu labelled" OR "menu labeling" OR "menu labelling"; anywhere in the article.

1. **OpenGrey**

Search string: (restaurant* OR cafeteria* OR canteen* OR "fast food" OR "vending machine*" OR menu* OR "food service") AND (label* OR post*) AND (calorie* OR kilojoule* OR energy OR nutri*)

1. **RIAN**

Search string: with any of ('"food services"', '"food service"', '"vending machines"', '"vending machine"', '"fast food"', 'restaurant', 'restaurants', 'cafeteria', 'cafeterias', 'canteen', 'canteens', 'menu', 'menus') AND ('label', 'labels', 'labelling', 'labeling', 'labelled', 'labeled', 'post', 'posts', 'posting', 'postings') AND ('calorie', 'calories', 'kilojoule', 'kilojoules', 'energy', 'nutrition', 'nutritional', 'nutrient', 'nutrients') in all fields

1. **EThOS**

Advanced search, abstract: (“food service” OR menu OR restaurant OR “fast food” OR canteen OR “vending machine”)

1. **ProQuest Dissertations & Theses**

Search string: ab,ti(restaurant* OR cafeteria* OR canteen* OR "fast food" OR "vending machine*" OR menu* OR "food service") AND ab,ti(label[*4] OR post[*4]) AND ab,ti(calorie* OR kilojoule* OR energy OR nutri[*6])

1. **WorldCat**

Search string: [ti:((restaurant* OR cafeteria* OR canteen* OR "fast food" OR "vending machine*" OR menu* OR "food service") AND (label* OR post*) AND (calorie* OR kilojoule* OR energy OR nutri*))](https://www.worldcat.org/search?q=kw%3A%28%28restaurant*+OR+cafeteria*+OR+canteen*+OR+%26quot%3Bfast+food%26quot%3B+OR+%26quot%3Bvending+machine*%26quot%3B+OR+menu*+OR+%26quot%3Bfood+service%26quot%3B%29+AND+%28label*+OR+post+OR+posts+OR+posting+OR+postings%29+AND+%28calorie*+OR+kilojoule*+OR+energy+OR+nutrition*+OR+nutrient*%29%29+ti%3A%28%28restaurant*+OR+cafeteria*+OR+canteen*+OR+%26quot%3Bfast+food%26quot%3B+OR+%26quot%3Bvending+machine*%26quot%3B+OR+menu*+OR+%26quot%3Bfood+service%26quot%3B%29+AND+%28label*+OR+post+OR+posts+OR+posting+OR+postings%29+AND+%28calorie*+OR+kilojoule*+OR+energy+OR+nutrition*+OR+nutrient*%29%29&qt=facetNavigation&dblist=638)

1. **Networked Digital Library of Theses and Dissertations**

Search string: description:(("food services" OR "food service" OR "vending machines" OR "vending machine" OR "fast food" OR restaurant OR restaurants OR cafeteria OR cafeterias OR canteen OR canteens OR menu OR menus) AND (label OR labels OR labelling OR labeling OR labelled OR labeled OR post OR posts OR posting OR postings) AND (calorie OR calories OR kilojoule OR kilojoules OR energy OR nutrition OR nutritional OR nutrient OR nutrients))

1. **Open Access Theses and Dissertations**

Search string: abstract:("food services" OR "food service" OR "vending machines" OR "vending machine" OR "fast food" OR restaurant OR restaurants OR cafeteria OR cafeterias OR canteen OR canteens OR menu OR menus) AND abstract:(label OR labels OR labelling OR labeling OR labelled OR labeled OR post OR posts OR posting OR postings) AND abstract:(calorie OR calories OR kilojoule OR kilojoules OR energy OR nutrition OR nutritional OR nutrient OR nutrients)

1. **Public health organisation websites**

Food Safety Authority of Ireland (website: www.fsai.ie); Food Standards Agency (website: www.food.gov.uk); Irish Health Service Executive (website: www.hse.ie); Department of Health (website: health.gov.ie); The Danish Cancer Society (website: [www.cancer.dk](http://www.cancer.dk)); NSW Food Authority (website: <http://www.foodauthority.nsw.gov.au>); Irish Nutrition & Dietetic Institute (website: www.indi.ie); Safefood (website: www.safefood.eu); Government of the United Kingdom (website: www.gov.uk); British Nutrition Foundation: (website: www.nutrition.org.uk); National Health Service (England) (website: www.nhs.uk); Center for Science in the Public Interest (website: https://cspinet.org); National Heart Foundation of Australia (website: www.heartfoundation.org.au); Food Standards Australia New Zealand (website: www.foodstandards.gov.au); Ontario Public Health Organisation (website: www.opha.on.ca); Ontario Ministry of Health and Long Term Care (website: www.health.gov.on.ca); Dietitians of Canada (website: www.dietitians.ca); U.S. Food and Drug Administration (website: www.fda.gov); American Heart Association (website: www.heart.org); Canadian Food Inspection Agency (website: www.inspection.gc.ca); British Dietetic Association (website: www.bda.uk.com); Academy of Nutrition and Dietetics (website: www.eatright.org); Dietitians Association of Australia (website: <https://daa.asn.au/>); Heart and Stroke Foundation of Canada (website: [www.heartandstroke.ca](http://www.heartandstroke.ca)); British Heart Foundation (website: [www.bhf.org.uk](http://www.bhf.org.uk)); Irish Heart Foundation (website: www.irishheart.ie)
