# Supplementary material for: Barriers and facilitators to implementation of menu labelling interventions from a food service industry perspective: a mixed methods systematic review
Source: Int J Behav Nutr Phys Act. 2020 Apr 15;17:48. doi: 10.1186/s12966-020-00948-1 (PMC7161210; doi:10.1186/s12966-020-00948-1)
Supplement: Supplementary file 3 — Additional file 3. Standardised data extraction form. This file provides the data extraction form used in this review. [file 12966_2020_948_MOESM3_ESM.docx]

**Additional file 3** Standardised data extraction form

First author

Publication year

Title

Country of origin

Publication type (e.g. peer-reviewed article or grey literature)

Publication language

Study aim

Study design

Study methods (e.g. quantitative, qualitative or mixed methods)

Data source (e.g. questionnaires, interviews)

Study participants (and sample size)

Setting (and sample size) (e.g. type of food service establishment)

Intervention type (e.g. quantitative – numeric or qualitative – interpretive menu labels)

Scheme (e.g. voluntary or mandatory)

Theory employed (if any)

Phase of implementation (e.g. pre, during or post-implementation)

Main findings (i.e. data on barriers and facilitators)
