# Supplementary material for: Barriers and facilitators to implementation of menu labelling interventions from a food service industry perspective: a mixed methods systematic review
Source: Int J Behav Nutr Phys Act. 2020 Apr 15;17:48. doi: 10.1186/s12966-020-00948-1 (PMC7161210; doi:10.1186/s12966-020-00948-1)
Supplement: Supplementary file 4 — Additional file 4. Final codebook and coding assumptions. This file provides the final codebook and coding assumptions used in this review. [file 12966_2020_948_MOESM4_ESM.docx]

**Additional file 4** Final codebook and coding assumptions

The codebook below is a results of (a) operationalisation/adaptation of existing definitions and eligibility criteria for CFIR constructs (available at [www.cfirguide.org](http://www.cfirguide.org)) to the topic area (i.e. menu labelling in the food service setting), (b) adaptation of existing construct definitions and eligibility criteria to improve coding consistency (i.e. to help distinguish constructs where unclear boundaries exist), (c) the addition of new coding assumptions to capture the interdependency of factors more accurately (e.g. ‘Engaging: External Change Agents’ and ‘Access to Knowledge & Information’ constructs) and (d) newly developed construct definitions and eligibility criteria (from the inductive analysis).

Coding Notes:

- Only code data that is expressed as a barrier or facilitator to implementation of menu labelling. A barrier is defined as any variable that impedes or obstructs the implementation of menu labelling. A facilitator is defined as any variable that eases and promotes the implementation of menu labelling.
- Code to the lowest level as indicated by the data (i.e. what is being explicitly said); thus, going with the least amount of inference or interpretation when coding.
- When coding data, remember the domain where the construct resides. For example, ‘Intervention Characteristics’ are perceived characteristics of the intervention regardless of context, while the ‘Inner Setting’ looks at the intervention within a specific context.
- Code to the construct that captures the theme more specifically.
- Code any relationships that arise in the data.
- Code general statements that do not specify the actual barrier or facilitator (e.g. “this is impractical and burdensome, so refuse to implement”) to ‘Knowledge & Beliefs about Menu Labelling’.

CFIR Domain Descriptions:

- Intervention Characteristics: Perceived characteristics of the intervention regardless of context.
- Outer Setting: External context of food service businesses (e.g. legislation, economy, consumers).
- Inner Setting: Internal context of food service businesses (e.g. level of staffing, resources).
- Characteristics of Individuals: Characteristics of the individuals involved in implementation (e.g. attitudes, beliefs).
- Process: Process involved in the implementation of menu labelling interventions.

| **Intervention Characteristics** | **Description** |
| --- | --- |
| 1. Intervention Source | Definition: Perception of stakeholders about whether the menu labelling intervention is externally or internally developed.  Inclusion Criteria: Include statements about the source of the intervention and the extent to which stakeholders viewed the introduction of this as internal or external to the food business.  Exclusion Criteria: Exclude statements related to who participated in the decision making process to implement menu labelling and code to ‘Engaging’, as an indication of early (or late) engagement. |
| 1. Evidence Strength & Quality | Definition: Stakeholders’ perceptions of the quality and validity of evidence supporting the belief that the menu labelling intervention will have desired outcomes.  Inclusion Criteria: Include statements regarding awareness of evidence and the strength and quality of evidence, as well as the absence of evidence or a desire for different types of evidence (e.g. consumer feedback) instead of evidence from the literature.  Exclusion Criteria: Exclude consumer feedback that does not relate to whether menu labelling is having the desired outcome and code to ‘Consumer Needs and Resources’. Exclude statements regarding the receipt of evidence as an engagement strategy and code to ‘[Engaging](http://cfirguide.org/wiki/index.php?title=Engaging)’. |
| 1. Relative Advantage | Definition: Stakeholders’ perception of the advantage of implementing menu labelling compared to the status quo or an alternative.  Inclusion Criteria: Include statements that demonstrate menu labelling is better (or worse) than the status quo or an alternative intervention. The underlying reason for ‘relative (dis)advantage’ may be captured in ‘Goals & Feedback’; double code when applicable.  Double code ‘Compatibility’, ‘Consumer Needs & Resources’ or ‘Economic Climate’ where it influences stakeholders’ perception of the (dis)advantage of implementing menu labelling compared to an alternative.  Exclusion Criteria: Exclude statements that do or do not demonstrate a strong need for menu labelling and/or that the current situation is untenable (e.g. statements that menu labelling is absolutely necessary or that menu labelling is redundant with other interventions) and code to ‘[Tension for Change](http://cfirwiki.net/wiki/index.php?title=Tension_for_Change)’. Exclude statements regarding specific needs of individuals (i.e. consumers) that demonstrate a need for menu labelling and code to ‘Consumer Needs & Requirements’. |
| 1. Adaptability | Definition: Perceived inherent ability/allowance to adapt the menu labelling intervention.  Inclusion Criteria: Include statements related to perceptions of the (in)ability to adapt the menu labelling intervention.  Exclusion Criteria: Exclude statements regarding actual or suggested adaptations to the menu labelling intervention to meet local needs and code to new construct ‘Adapting’ under the CFIR ‘Process’ domain. |
| 1. Trialability | Definition: Perceived inherent ability/allowance to trial the menu labelling intervention on a small scale, and to be able to reverse course (undo implementation) if warranted.  Inclusion Criteria: Include statements related to perceptions of the (in)ability to trial (i.e. test/pilot) the menu labelling intervention.  Exclusion Criteria: Exclude statements regarding actual or suggested trials of the menu labelling intervention and code to new construct ‘Trialing’ under the CFIR ‘Process’ domain. Exclude descriptions of use of results from pilots and code to ‘[Evidence Strength & Quality](http://cfirwiki.net/wiki/index.php?title=Evidence_Strength_%26_Quality)’. |
| 1. Complexity | Definition: Perceived difficulty of menu labelling, reflected by duration, scope, radicalness, disruptiveness, centrality, and intricacy and number of steps required to implement.  Inclusion Criteria: Code statements regarding the complexity of the menu labelling intervention, which are not dependent on context.  Exclusion Criteria: Exclude statements which refer to factors operating within the internal setting of food businesses which effect implementation (e.g. lack of standardised recipes, lack of time) and code to relevant construct under the ‘Inner Setting’ domain. |
| 1. Design Quality & Packaging | Definition: Perceived excellence in how menu labelling is bundled, presented, and assembled.  Inclusion Criteria: Include statements regarding the quality of the materials and packaging used in menu labelling. Packaging relates to how menu labelling is presented and even how accessible it is for users (i.e. consumers).  Exclusion Criteria: Exclude statements regarding the presence or absence of materials and code to ‘[Available Resources](http://cfirwiki.net/wiki/index.php?title=Available_Resources)’. Exclude statements regarding the receipt of materials as an engagement strategy and code to ‘[Engaging](http://cfirwiki.net/wiki/index.php?title=Engaging)’. |
| 1. Cost | Definition: Costs of the intervention and costs associated with implementing the intervention.  Inclusion Criteria: Include statements related to the cost of menu labelling and its implementation.  Double code ‘Structural Characteristics’ where it influences cost.  Exclusion Criteria: Exclude statements which refer to the presence or absence of resources in the internal setting of food businesses and code to ‘Available Resources’ (e.g. money, time). |
| **Outer Setting** |  |
| 1. Consumer Needs & Resources | Definition: The extent to which the needs of consumers, as well as barriers and facilitators to meet those needs, are accurately known and prioritized by the food business.  Inclusion Criteria: Include statements demonstrating (lack of) awareness of consumer needs and resources (e.g. consumer demand for menu labelling, barriers and facilitators of consumer participation in menu labelling, consumer satisfaction with menu labelling).  Exclusion Criteria: Exclude consumer feedback on whether menu labelling is having the desired outcome and code to ‘Evidence Strength & Quality’. |
| 1. Cosmopolitanism | Definition: The degree to which the food business is networked with other external organizations (i.e. external people and groups).  Inclusion Criteria: Include descriptions of outside group memberships and networking done outside the food business, which are independent of the menu labelling intervention.  Exclusion Criteria: Exclude statements of networking with external organisations that did not exist prior to the menu labelling intervention and code to ‘Engaging’ construct. Exclude statements about general networking, communication, and relationships in the food business, which are independent of the menu labelling intervention and code to ‘[Networks & Communications](http://cfirwiki.net/wiki/index.php?title=Networks_%26_Communications)’. |
| 1. Peer Pressure | Definition: Mimetic or competitive pressure to implement menu labelling; typically, because most or other key peer or competing food businesses have already implemented or are in a bid for a competitive edge.  Inclusion Criteria: Include statements about perceived pressure or motivation from other food businesses to implement menu labelling. |
| 1. Media & Societal Pressure* | Definition: Perceived pressure from media and societal expectations/watch-dogging.  Inclusion Criteria: Include statements about perceived pressure or motivation from media or society (i.e. mass media, the community, the public).  Exclusion Criteria: Exclude statements about perceived pressure or motivation from other food businesses and code to ‘Peer Pressure’. Exclude statements about perceived pressure or motivation from consumers and code to ‘Consumer Needs & Resources’. |
| 1. External Policy & Incentives | Definition: A broad construct that includes external strategies to spread menu labelling, including policy and regulations (governmental or other central entity), external mandates, recommendations and guidelines and public reporting.  Inclusion Criteria: Include descriptions of external strategies (outside the food business) to spread menu labelling (e.g. policies, regulations, guidelines). |
| 1. Economic Climate* | Definition: The extent to which the economic climate is known and prioritised by the food business.  Inclusion Criteria: Include statements demonstrating awareness of the economic climate and its influence on implementation of menu labelling.  Exclusion Criteria: Exclude statements which refer to the presence or absence of resources in the internal setting of food businesses and code to ‘Available Resources’ (e.g. money). |
| 1. Educational System* | Definition: A broad construct which refers to the educational system.  Inclusion Criteria: Include statements about the educational system and its influence on implementation of menu labelling.  Exclusion Criteria: Exclude statements which refer to access to knowledge and information about menu labelling within the food business and code to ‘[Access to Knowledge & Information](http://cfirwiki.net/wiki/index.php?title=Access_to_Knowledge_%26_Information). |
| **Inner Setting** |  |
| 1. Structural Characteristics | Definition: The social architecture, age, maturity, and size of the food business.  Inclusion Criteria: Include statements about the size, age, maturity and degree of specialisation of the food business. Furthermore, include statements on decision-making autonomy, staff turnover and ratio of managers to total employees in the food business. |
| 1. Networks & Communications | Definition: The nature and quality of webs of social networks and the nature and quality of formal and informal communications within the food business.  Inclusion Criteria: Include statements about general networking, communication, and relationships in the food business (such as descriptions of meetings, email groups, or other methods of keeping people connected and informed) and statements related to team formation, quality, and functioning – needs to be independent of the menu labelling intervention.  Exclusion Criteria: Exclude statements about networking and communication that did not exist prior to the menu labelling intervention and code to ‘Engaging’ construct. Exclude statements related to implementation leaders' and staffs access to knowledge and information regarding using menu labelling and code to ‘[Access to Knowledge & Information](http://cfirwiki.net/wiki/index.php?title=Access_to_Knowledge_%26_Information)’. Exclude descriptions of outside group memberships and networking done outside the food business, which are independent of the menu labelling intervention and code to ‘[Cosmopolitanism](http://cfirwiki.net/wiki/index.php?title=Cosmopolitanism)’. |
| 1. Culture | Definition: Norms, values, and basic assumptions of the food business.  Note: Culture is often viewed as relatively stable, socially constructed, and subconscious.  Inclusion Criteria: Include statements related to concepts captured in the Competing Values Framework approach - four archetypical organizational cultures: team culture, hierarchical culture, entrepreneurial culture and rational culture. |
| 1. Implementation Climate | Definition: The absorptive capacity for change, shared receptivity of involved individuals to the menu labelling intervention, and the extent to which use of that intervention will be rewarded, supported, and expected within their food business.  Inclusion Criteria: Include statements regarding the general level of receptivity to implementing menu labelling.  Exclusion Criteria: Exclude statements regarding the general level of receptivity that are captured in the sub-codes below. |
| 1. Tension for change | Definition: The degree to which stakeholders perceive the current situation as intolerable or needing change.  Inclusion Criteria: Include statements that (do not) demonstrate a strong need for menu labelling and/or that the current situation is untenable (e.g. statements that the menu labelling is absolutely necessary or that it is redundant with other programs).  Double code statements to ‘Consumer Needs & Resources’ where consumer needs and preferences is driving the need for menu labelling.  Exclusion Criteria: Exclude statements that demonstrate the intervention is better (or worse) than existing programs and code to ‘[Relative Advantage](http://cfirwiki.net/wiki/index.php?title=Relative_Advantage)’. |
| 1. Compatibility | Definition: The degree of tangible fit between meaning and values attached to menu labelling by involved individuals, how those align with individuals’ own norms, values, and perceived risks and needs, and how menu labelling fits with existing workflows and systems.  Inclusion Criteria: Include statements that demonstrate the level of compatibility menu labelling has with the food business mission/purpose/values and ways of working.  Double code ‘Structural Characteristics’ or ‘Consumer Needs & Resources’ where it influences compatibility with organisation work processes. |
| 1. Relative Priority | Definition: Individuals’ shared perception of the importance of implementing menu labelling within the food business.  Inclusion Criteria: Include statements that reflect the relative priority of menu labelling (e.g. statements related to change fatigue in the food business due to implementation of many other programs).  Double code ‘Available Resources’ or ‘Economic Climate’ where it influences the relative priority of menu labelling. |
| 1. Incentives & Rewards | Definition: Extrinsic incentives such as goal-sharing awards, performance reviews, promotions, and raises in salary, and less tangible incentives such as increased stature or respect.  Inclusion Criteria: Include statements related to whether incentive systems are in place to foster (or hinder) implementation, e.g. rewards or disincentives for staff engaging in menu labelling. |
| 1. Goals & Feedback | Definition: The degree to which goals are clearly communicated, acted upon, and fed back to staff, and alignment of that feedback with goals.  Inclusion Criteria: Include statements related to the (lack of) alignment of menu labelling with larger goals of the food business, as well as feedback to staff regarding those goals. Goals can be related to (un)stated food business needs (e.g. profitability).  Double code statements to ‘Structural Characteristics’ or ‘Consumer Needs & Resources’ where it influences organisational goals and feedback.  Note: ‘Goals and Feedback’ is independent of the implementation process; it likely continues when implementation activities end.  Exclusion Criteria: Exclude statements that refer to the process used in implementation (i.e. the implementation team’s (lack of) on-going review of implementation progress and code to ‘[Reflecting & Evaluating](http://cfirguide.org/wiki/index.php?title=Reflecting_%26_Evaluating)’). ‘Reflecting and Evaluating’ is part of the implementation process; it likely ends when implementation activities end. |
| 1. Learning Climate | Definition: A climate in which: a) leaders express their own fallibility and need for team members’ assistance and input; b) team members feel that they are essential, valued, and knowledgeable partners in the change process; c) individuals feel psychologically safe to try new methods; and d) there is sufficient time and space for reflective thinking and evaluation.  Inclusion Criteria: Include statements that support (or refute) the degree to which key components of the food business exhibit a ‘learning climate’. |
| 1. Readiness for Implementation | Definition: Tangible and immediate indicators of food business commitment to its decision to implement menu labelling.  Inclusion Criteria: Include statements regarding the general level of readiness for implementation.  Exclusion Criteria: Exclude statements regarding the general level of readiness for implementation that are captured in the sub-codes below. |
| 1. Leadership engagement | Definition: Commitment, involvement, and accountability of leaders and managers with the implementation.  Inclusion Criteria: Include statements regarding the level of commitment/support of leadership in the food business.  Double code statements regarding leadership engagement to ‘Engaging: Opinion Leader’, ‘Engaging: [Formally Appointed Internal Implementation Leaders](http://cfirwiki.net/wiki/index.php?title=Formally_Appointed_Internal_Implementation_Leaders)’ or ‘Engaging: [Champions](http://cfirwiki.net/wiki/index.php?title=Champions)’ if the food business leader is also an implementation leader (e.g. if a director of food outlet takes the lead in implementing menu labelling). Note that a key characteristic of this Implementation Leader/Champion or Opinion Leader is that s/he is also a leader in the food business. |
| 1. Available Resources | Definition: The level of resources dedicated for implementation and on-going operations, including money, training, education, physical space, and time.  Inclusion Criteria: Include statements related to the presence or absence of resources described above or resources specific to menu labelling implementation.  Double code statements to ‘Structural Characteristics’ where it influences the level of available resources.  Exclusion Criteria: Exclude statements related to training and education and code to the CFIR sub-construct ‘[Access to Knowledge & Information](http://cfirwiki.net/wiki/index.php?title=Access_to_Knowledge_%26_Information)’. Exclude statements related to the quality of materials and code to the CFIR construct ‘[Design Quality & Packaging](http://cfirwiki.net/wiki/index.php?title=Design_Quality_%26_Packaging)’. |
| 1. Access to Knowledge & Information | Definition: Ease of access to digestible information and knowledge about menu labelling and how to incorporate it into work tasks.  Note: Information and knowledge includes all sources such as experts, other experienced staff, training, documentation, and computerized information systems.  Inclusion Criteria: Include statements related to stakeholder access to knowledge and information regarding the menu labelling intervention. Include knowledge and information available from within the implementing food business (e.g. training provided by staff) or those available from external entities/stakeholders to the food business (e.g. via a health agency or supplier providing information).  Double code statements to the CFIR sub-construct ‘Engaging: External Change Agent’ or the new construct ‘Engaging: External Key Stakeholder’ where access to knowledge and information is provided by an external entity/stakeholder to facilitate implementation.  Double code ‘Structural Characteristics’ or ‘Available Resources’ where it influences access to knowledge and information.  Exclusion Criteria: Exclude statements about general networking, communication, and relationships in the organisation, which are independent of the menu labelling intervention and code to ‘[Networks & Communications](http://cfirwiki.net/wiki/index.php?title=Networks_%26_Communications)’. |
| **Characteristics of Individuals** |  |
| 1. Knowledge & Beliefs about Menu Labelling | Definition: Individuals’ attitudes toward and value placed on menu labelling as well as familiarity with facts, truths, and principles related to the intervention.  Double code ‘Structural Characteristics’ where it influences knowledge and beliefs about menu labelling.  Exclusion Criteria: Exclude statements related to familiarity with the evidence regarding menu labelling and code to ‘[Evidence Strength & Quality](http://cfirwiki.net/wiki/index.php?title=Evidence_Strength_%26_Quality)’. |
| 1. Self-efficacy | Definition: Individual belief in their own capabilities (confidence in their ability) to execute courses of action to achieve implementation goals (i.e. to carry out steps required to implement menu labelling). |
| 1. Individual Stage of Change | Definition: Characterisation of the phase an individual is in, as he or she progresses toward skilled, enthusiastic, and sustained use of menu labelling. |
| 1. Individual Identification with the Food Business | Definition: A broad construct related to how individuals perceive the food business, and their relationship and degree of commitment with that business. |
| 1. Other Personal Attributes | Definition: A broad construct to include other personal traits such as tolerance of ambiguity, intellectual ability, motivation, values, competence, capacity, and learning style. |
| **Process** |  |
| 1. Planning | Definition: The degree to which a scheme or sequence of tasks for implementing menu labelling are developed in advance, and the quality of those schemes or tasks.  Inclusion Criteria: Include evidence of pre-implementation diagnostic assessments and planning, as well as refinements to the plan. |
| 1. Engaging | Definition: Attracting and involving appropriate individuals in the implementation and use of the intervention through a combined strategy of social marketing, education, role modelling, training, and other similar activities.  Inclusion Criteria: Include statements related to engagement strategies and outcomes (i.e. if and how stakeholders became engaged with menu labelling and what their role is in implementation).  Exclusion Criteria: Exclude statements that are captured in the sub-codes below. |
| 1. Opinion Leaders | Definition: Individuals in the food business who have formal or informal influence on the attitudes and beliefs of their colleagues with respect to implementing menu labelling.  Note: There is general agreement that there are two different types of opinion leaders, experts and peers. Expert opinion leaders exert influence through their authority and status. Peer opinion leaders exert influence through their representativeness and credibility.  Inclusion Criteria: Include statements related to engagement strategies and outcomes (e.g. how the opinion leader became engaged with menu labelling and what their role is in implementation).  Double code statements to the CFIR sub-construct ‘Leadership Engagement’ (under the ‘Inner Setting’ domain) if the opinion leader is also a food business leader/manager. |
| 1. Formally Appointed Internal Implementation Leader | Definition: Individuals from within the food business who have been formally appointed with responsibility for implementing menu labelling as coordinator, project manager, team leader, or other similar role.  Inclusion Criteria: Include statements related to engagement strategies and outcomes (e.g. how the formally appointed internal implementation leader became engaged with menu labelling and what their role is in implementation).  Double code statements to the CFIR sub-construct ‘Leadership Engagement’ (under the ‘Inner Setting’ domain) if the formally appointed internal implementation leader is also a food business leader/manager (e.g. if a director of food business takes the lead in implementing menu labelling). |
| 1. Champions | Definition: Individuals who dedicate themselves to supporting, marketing, and ‘driving through’ an implementation, overcoming indifference or resistance that a menu labelling intervention may provoke in a food business.  Inclusion Criteria: Include statements related to engagement strategies and outcomes (e.g. how the champion became engaged with the menu labelling intervention and what their role is in implementation).  Double code statements to the CFIR sub-construct ‘Leadership Engagement’ (under the ‘Inner Setting’ domain) if the champion is also a food business leader/manager. |
| 1. External Change Agents | Definition: Individuals who are affiliated with an outside entity (related or unrelated to the food business) who formally influence or facilitate menu labelling decisions in a desirable direction.  External change agents may include researchers, health agency staff, health professionals and hired consultants external to the food business.  Inclusion Criteria: Include statements related to engagement strategies and outcomes (e.g. how the external change agent became engaged with menu labelling and what their role is in implementation (e.g. how they supported implementation efforts).  Double code ‘Access to Knowledge & Information’ where the external change agent influences access to knowledge and information.  Double code ‘Available Resources’ where it influences the ability to engage external change agents. |
| 1. Internal Key Stakeholders* | Definition: Individuals from within the food business with responsibility for implementing menu labelling.  Note: Internal key stakeholders may include chef, waiters, serving personnel etc.  Inclusion Criteria: Include statements related to engagement strategies and outcomes, e.g., how internal key stakeholders became engaged with the innovation and what their role was in implementation.  Exclusion Criteria: Exclude statements related to internal stakeholders who act as a champion for menu labelling and code ‘Engaging: Champions’. Exclude statements related to internal stakeholders who have been formally appointed as implementation leaders and code to ‘Formally Appointed Internal Implementation Leader’. |
| 1. External Key Stakeholders* | Definition: Individuals from outside the food business with an indirect role in implementing menu labelling.  Note: External key stakeholder may include food suppliers/purveyors.  Inclusion Criteria: Include statements related to engagement strategies and outcomes, e.g., how external key stakeholders became engaged with the innovation and what their role is in implementation.  Double code ‘Access to Knowledge & Information’ where the external change agent provides knowledge and information to the food business.  Exclusion Criteria: Exclude statements related to external change agents and code to ‘External Change Agents’. |
| 1. Executing | Definition: Carrying out or accomplishing the implementation according to plan.  Inclusion Criteria: Include statements that demonstrate how implementation occurred with respect to the implementation plan.  Note: Fidelity measures can be used to assess executing, as an indication of the degree to which implementation was accomplished according to plan. |
| 1. Reflecting & Evaluating | Definition: Quantitative and qualitative feedback about the progress and quality of implementation accompanied with regular personal and team debriefing about progress and experience.  Inclusion Criteria: Include statements that refer to the implementation team’s (lack of) assessment of the progress toward and impact of implementation, as well as the interpretation of outcomes related to implementation.  Note: ‘Reflecting and Evaluating’ is part of the implementation process; it likely ends when implementation activities end. It does not require goals be explicitly articulated; it can focus on descriptions of the current state with real-time judgment, though there may be an implied goal (e.g. we need to implement menu labelling) when the implementation team discusses feedback in terms of adjustments needed to complete implementation.  Evidence of the integration of evaluation components used as part of ‘Reflecting and Evaluating’ into on-going or sustained organizational structures and processes may be double coded to ‘Goals and Feedback’.  Exclusion Criteria: Exclude statements related to the (lack of) alignment of menu labelling goals with larger food business goals, as well as feedback to staff regarding those goals and code to ‘[Goals & Feedback](http://cfirwiki.net/wiki/index.php?title=Goals_%26_Feedback)’. |
| 1. Adapting the Organisation* | Definition: Activities which are related to refining existing workflows to accommodate the menu labelling intervention (i.e. adapting the organisation).  Inclusion Criteria: Include statements which describe activities to refine existing work practices to accommodate menu labelling. |
| 1. Adapting the Intervention* | Definition: Actual or suggested adaptations to the menu labelling intervention to meet local needs (i.e. adapting the intervention).  Inclusion Criteria: Include statements which describe actual or suggested adaptations to the menu labelling intervention to meet local needs.  Double code statements to ‘Design Quality & Packaging’, ‘Compatibility’, ‘Consumer Needs & Resources’ or ‘External Policy & Incentives’ where it drives actual or suggested adaptations to the intervention.  Exclusion Criteria: Exclude statements related to perceptions of the (in)ability to adapt the menu labelling intervention and code to ‘Adaptability’. |
| 1. Trailing* | Definition: Actual or suggested trials of the menu labelling intervention.  Inclusion Criteria: Include statements which describe actual or suggested trials (i.e. testing/piloting) of the menu labelling intervention.  Exclusion Criteria: Exclude statements related to perceptions of the (in)ability to trial the menu labelling intervention and code to ‘Trialability’. |
| 1. Scaling Up* | Definition: Activities to assist with scaling up the menu labelling intervention.  Double code ‘Compatibility’ where it influences activities to assist with scaling up the intervention.  Inclusion Criteria: Include statements which describe activities undertaken to scale up the menu labelling intervention within the organisation. |

* new constructs generated inductively
