# Supplementary material for: Barriers and facilitators to implementation of menu labelling interventions from a food service industry perspective: a mixed methods systematic review
Source: Int J Behav Nutr Phys Act. 2020 Apr 15;17:48. doi: 10.1186/s12966-020-00948-1 (PMC7161210; doi:10.1186/s12966-020-00948-1)
Supplement: Supplementary file 5 — Additional file 5. Characteristics of included studies. This file provides information on the characteristics of included studies in this review. [file 12966_2020_948_MOESM5_ESM.docx]

**Additional file 5** Characteristics of included studies (*n*=17)

| **Author, year, country of origin** | **Publication type** | **Design** | **Methods, data source** | **Study participants (*n*)** | **Setting** | **Intervention type** | **MMAT score, %** |
| --- | --- | --- | --- | --- | --- | --- | --- |
| Almanza, 1997, USA [[1](#_ENREF_1)] | Journal article | Cross-sectional | Quantitative.  Questionnaire | Research and development directors (*n*=65) | Large foodservice corporations (*n* not reported) | Not specified | 50 |
| Britt, 2011, USA [[2](#_ENREF_2)] | Journal article | Cross-sectional | Qualitative.  Interviews | Managers/owners (*n* not reported) | 9 independently owned restaurants | Numeric – calorie, fat, carbohydrate, & sodium information | 0 |
| Clegg, 2009, UK [[3](#_ENREF_3)] | Research report | Cross-sectional | Qualitative.  Interviews | Head office representatives and outlet managers (*n* not reported) | 21 catering outlets (includes seated service, counter service, self-selection and workplace catering) | Numeric – calorie information | 75 |
| Din, 2012, Malaysia [[4](#_ENREF_4)] | Journal article | Cross-sectional | Qualitative.  Interviews | Managers/owners (*n*=8) | 8 independently owned full service restaurants | Not specified | 25 |
| FSAI, 2012, Ireland [[5](#_ENREF_5)] | Research report | Cross-sectional | Quantitative.  Survey | Participants (type unknown) from foodservice businesses (*n*=199) | Foodservice businesses (majority responsible for 1-5 food outlets) | Numeric – calorie information | 25 |
| Geaney, 2015, Ireland [[6](#_ENREF_6)] | Research report | Cross-sectional | Quantitative & qualitative.  Survey & interviews | Managers/owners (*n*=604) - survey  Manager/owners (*n*=13) - interviews | 604 foodservice businesses (82 chains & 522 single outlet) - survey.  13 foodservice businesses (6 chain & 7 single outlet) – interviews. | Numeric – calorie information | 50 |
| Jeong Jin-Yi, 2015, South Korea [[7](#_ENREF_7)] | Journal article | Cross-sectional | Quantitative.  Questionnaire | Managers/owners (*n*=205) | 205 foodservice businesses | Numeric – calorie, protein, sodium, sugar & saturated fat information | 50 |
| Logue, 2013, Ireland [[8](#_ENREF_8)] | Conference proceeding | Cross-sectional | Quantitative.  Survey | Foodservice businesses participants (*n*=480) | 480 foodservice businesses (177 fast food, 115 restaurant, 77 coffee shop/deli, 17 catering companies) | Numeric – calorie information | 50 |
| Maestro, 2008, Brazil [[9](#_ENREF_9)] | Journal article | Cross-sectional | Quantitative.  Questionnaire | Managers (*n*=114) | 20 fast food restaurants and 94 full service restaurants | Not specified | 25 |
| Mah, 2013, Canada [[10](#_ENREF_10)] | Journal article | Cross-sectional | Quantitative & qualitative.  Survey, interviews & policy consultation | Managers/owners *(n*=256) – survey  Executives/decision makers (*n*=9) – interviews  Members of restaurant associations (*n* not reported) – policy consultation | 256 independent restaurants – survey  9 chain and franchise restaurants (6 large and 3 small, including quick-service and sit-down restaurants) – interviews  2 provincial restaurant associations – policy consultation | Not specified | 50 |
| Ottawa Public Health, 2013, Canada [[11](#_ENREF_11)] | Research report | Cross-sectional | Quantitative.  Survey | Managers/owners/supervisors (*n*=200) | 200 foodservice businesses (146 independent and 54 franchised operators) | Not specified | 50 |
| Ray, 2013, UK [[12](#_ENREF_12)] | Research report | Longitudinal | Qualitative.  Interviews & workshop discussions | Head office representatives and outlet managers (*n* not reported) | 9 food businesses taking part in Caloriewise (3 contract caterers, 3 hospital trusts, 1 sandwich shop chain, 1 commercial public house business & 1 restaurant serving ethnic cuisine) and food businesses participating in the Responsibility Deal pledge | Numeric – calorie information | 75 |
| Roodenburg, 2013, Netherlands [[13](#_ENREF_13)] | Conference proceeding | Cross-sectional | Qualitative.  Data source not specified | Managers, chefs and serving personnel (*n* not reported) | 4 restaurants | Not specified | 0 |
| Shupe, 2013, USA [[14](#_ENREF_14)] | Doctoral dissertation | Cross-sectional | Quantitative.  questionnaire | Managers (*n*=68) | 68 independent restaurants | Not specified | 50 |
| Toronto Public Health, 2015, Canada [[15](#_ENREF_15)] | Research report | Longitudinal | Qualitative.*  Interviews | Owners/staff (*n* not reported) | 22 independently owned/operated restaurants | Numeric – calorie & sodium information | 50 |
| Vyth, 2011, Netherlands [[16](#_ENREF_16)] | Journal article | Cross-sectional | Quantitative.  Questionnaire | Catering managers (*n*=316) | 2 large catering companies in worksite cafeterias | Interpretive – logo | 50 |
| Zick, 2010, UK [[17](#_ENREF_17)] | Journal article | Longitudinal | Qualitative.  Observations & interviews | Restaurant staff - nutritionist, manager, head chef & waiters (*n* not reported) | A 5-star hotel restaurant | Numeric – calorie, saturated fat, polyunsaturated fat, fibre & sodium information | 50 |

*Qualitative data extracted from a mixed methods study

**References**

1. Almanza BA, Nelson D, Chai S. Obstacles to nutrition labeling in restaurants. J Am Diet Assoc. 1997;97:157-61.

2. Britt JW, Frandsen K, Leng K, Evans D, Pulos E. Feasibility of voluntary menu labeling among locally owned restaurants. Health Promot Pract. 2011;12:18-24.

3. Clegg S, Jordan E, Slade Z. An Evaluation of Provision of Calorie Information by Catering Outlets. United Kingdom: Food Standards Agency, 2009.

4. Din N, Zahari MSM, Othman CN, Abas R. Restaurant operator's receptiveness towards providing nutritional information on menu. Procedia Soc Behav Sci 2012;50:699-709.

5. Food Safety Authority of Ireland (FSAI). Calories on menus in Ireland. A report on a national consultation. Dublin: FSAI; 2012.

6. Geaney F, Kelly C, Scotto Di Marrazzo J, Gilgan L, McCarthy M, Perry IJ. Evaluation of the uptake of voluntary calorie posting on menus in Ireland. Dublin: Department of Health, 2015.

7. Jeong JY, Kim E, Yang IS, Ham S. Motivators and Barriers to Provision of Nutritional Information in Restaurants. Korean Journal of Hospitality & Tourism 2015;24:227-43.

8. Logue D, Kennelly J, Keaveney E, O’Connor D, Bhriain SN, Flynn M. Calorie menu labelling in Ireland: a survey of food service businesses. Proc Nutr Soc. 2013;72.

9. Maestro V, Salay E. Restaurant nutrition and health information in the municipality of Campinas, São Paulo, Brazil: expectations of managers with respect to benefits and obstacles. Journal of Foodservice. 2008;19:262-9.

10. Mah CL, Vanderlinden L, Mamatis D, Ansara DL, Levy J, Swimmer L. Ready for policy? Stakeholder attitudes toward menu labelling in Toronto, Canada. Can J Public Health. 2013;104:e229-34.

11. Ottawa Public Health. Report on Ottawa Restaurant Survey. Ottawa: Ottawa Public Health; 2013.

12. Ray K, Clegg S, Davidson R, Vegeris S. Evaluation of Caloriewise: A Northern Ireland pilot of the display of calorie information in food catering businesses. Northern Ireland: Food Standards Agency, 2013.

13. Roodenburg AC, Payens IJ, Vrijhof C. Menu labeling in “out-of-home” sector: opportunities, barriers, and needs with respect to use of health communication in restaurants. Ann Nutr Metab. 2013;63(suppl1):1054.

14. Shupe E. Obstacles to Participation in Menu Labeling Observed by the Independent Foodservice Establishments [Degree of Doctor of Philosophy]. Minnesota, United States: Walden University; 2013.

15. Toronto Public Health. Voluntary Menu Labelling Pilot Project: Final Report. Ontario, Canada: Toronto Public Health; 2015.

16. Vyth EL, Van Der Meer EW, Seidell JC, Steenhuis IH. A nutrition labeling intervention in worksite cafeterias: an implementation evaluation across two large catering companies in the Netherlands. Health Promotion Int. 2011;27:230-7.

17. Zick A, Wake Y, Reeves S. Nutrition labelling in restaurants: a UK-based case study. NUFS. 2010;40:557-65.
