# Supplementary material for: Barriers and facilitators to implementation of menu labelling interventions from a food service industry perspective: a mixed methods systematic review
Source: Int J Behav Nutr Phys Act. 2020 Apr 15;17:48. doi: 10.1186/s12966-020-00948-1 (PMC7161210; doi:10.1186/s12966-020-00948-1)
Supplement: Supplementary file 6 — Additional file 6. Quality assessment of each study using MMAT criteria. This file provides a detailed breakdown of the quality assessment for each study. [file 12966_2020_948_MOESM6_ESM.docx]

| **Additional file 6** Quality assessment of each study using MMAT criteria | | | | | | | | | | | | | | | | | |
| --- | --- | --- | --- | --- | --- | --- | --- | --- | --- | --- | --- | --- | --- | --- | --- | --- | --- |
|  | Britt, 2011 [[1](#_ENREF_1)] | Clegg, 2009 [[2](#_ENREF_2)] | Din, 2012 [[3](#_ENREF_3)] | Ray, 2013 [[4](#_ENREF_4)] | Roodenburg, 2013 [[5](#_ENREF_5)] | Toronto Public Health, 2015 [[6](#_ENREF_6)] | Zick, 2010 [[7](#_ENREF_7)] | Geaney,2015 [[8](#_ENREF_8)] | Mah, 2013 [[9](#_ENREF_9)] | Almanza, 1997 [[10](#_ENREF_10)] | FSAI, 2012 [[11](#_ENREF_11)] | Jeong Jin-Yi, 2015 [[12](#_ENREF_12)] | Logue, 2013 [[13](#_ENREF_13)] | Maestro, 2008 [[14](#_ENREF_14)] | Ottawa Public Health, 2013 [[15](#_ENREF_15)] | Shupe, 2013 [[16](#_ENREF_16)] | Vyth, 2011 [[17](#_ENREF_17)] |
| **Screening questions** |  |  |  |  |  |  |  |  |  |  |  |  |  |  |  |  |  |
| Clear qualitative, quantitative or mixed methods research question? | ✘ | ✔ | ✔ | ✔ | ✔ | ✔ | ✔ | ✔ | ✔ | ✔ | ✔ | ✔ | ✔ | ✔ | ✔ | ✔ | ✔ |
| Collected data address the research question? | **ʘ** | ✔ | ✔ | ✔ | **ʘ** | ✔ | ✔ | ✔ | ✔ | ✔ | ✔ | ✔ | ✔ | ✔ | ✔ | ✔ | ✔ |
| **Qualitative** |  |  |  |  |  |  |  |  |  |  |  |  |  |  |  |  |  |
| Sources of qualitative data relevant to address research question? | **ʘ** | ✔ | ✔ | ✔ | **ʘ** | ✔ | ✔ | ✔ | ✔ |  |  |  |  |  |  |  |  |
| Process for analysing qualitative data relevant to address research question? | **ʘ** | ✔ | **ʘ** | ✔ | **ʘ** | **ʘ** | **ʘ** | ✔ | ✔ |  |  |  |  |  |  |  |  |
| Appropriate consideration given to how findings relate to the context in which data collected? | ✘ | ✔ | ✘ | ✔ | ✘ | ✔ | ✔ | ✘ | ✘ |  |  |  |  |  |  |  |  |
| Appropriate consideration given to how findings relate to researchers’ influence? | ✘ | ✘ | ✘ | ✘ | ✘ | ✘ | ✘ | ✘ | ✘ |  |  |  |  |  |  |  |  |
| **Quantitative descriptive** |  |  |  |  |  |  |  |  |  |  |  |  |  |  |  |  |  |
| Is sampling strategy relevant to address research question? |  |  |  |  |  |  |  | ✔ | ✔ | ✔ | ✔ | **ʘ** | **ʘ** | ✔ | ✔ | ✔ | ✔ |
| Is the sample representative of the population under study? |  |  |  |  |  |  |  | ✔ | **ʘ** | **ʘ** | **ʘ** | **ʘ** | ✔ | **ʘ** | ✔ | ✘ | **ʘ** |
| Are measurements appropriate (clear origin, or validity know, or standard instrument)? |  |  |  |  |  |  |  | ✔ | ✔ | ✔ | **ʘ** | ✔ | **ʘ** | ✘ | **ʘ** | ✔ | ✔ |
| Is there an acceptable response rate (60% or above?) |  |  |  |  |  |  |  | ✔ | ✘ | ✘ | **ʘ** | ✔ | ✔ | **ʘ** | ✘ | ✘ | ✘ |
| **Mixed methods** |  |  |  |  |  |  |  |  |  |  |  |  |  |  |  |  |  |
| Is the mixed methods research design relevant to address the qualitative and quantitative research questions? |  |  |  |  |  |  |  | ✔ | ✔ |  |  |  |  |  |  |  |  |
| Is the integration of qualitative and quantitative data (or results) relevant to address the research question? |  |  |  |  |  |  |  | **ʘ** | ✘ |  |  |  |  |  |  |  |  |
| Is appropriate consideration given to limitations associated with integration? |  |  |  |  |  |  |  | ✘ | ✘ |  |  |  |  |  |  |  |  |
| **Quality Score** | 0% | 75% | 25% | 75% | 0% | 50% | 50% | 50% | 50% | 50% | 25% | 50% | 50% | 25% | 50% | 50% | 50% |
| ✔ = yes, ✘ = no, **ʘ** = can’t tell  MMAT 0% = very low quality, MMAT 25% = low quality, MMAT 50% = average quality, MMAT 75% = high quality, MMAT 100% = very high quality | | | | | | | | | | | | | | | | | |

**References**

1. Britt JW, Frandsen K, Leng K, Evans D, Pulos E. Feasibility of voluntary menu labeling among locally owned restaurants. Health Promot Pract. 2011;12:18-24.

2. Clegg S, Jordan E, Slade Z. An Evaluation of Provision of Calorie Information by Catering Outlets. United Kingdom: Food Standards Agency, 2009.

3. Din N, Zahari MSM, Othman CN, Abas R. Restaurant operator's receptiveness towards providing nutritional information on menu. Procedia Soc Behav Sci 2012;50:699-709.

4. Ray K, Clegg S, Davidson R, Vegeris S. Evaluation of Caloriewise: A Northern Ireland pilot of the display of calorie information in food catering businesses. Northern Ireland: Food Standards Agency; 2013.

5. Roodenburg AC, Payens IJ, Vrijhof C. Menu labeling in “out-of-home” sector: opportunities, barriers, and needs with respect to use of health communication in restaurants. Ann Nutr Metab. 2013;63(suppl1):1054.

6. Toronto Public Health. Voluntary Menu Labelling Pilot Project: Final Report. Ontario, Canada: Toronto Public Health; 2015.

7. Zick A, Wake Y, Reeves S. Nutrition labelling in restaurants: a UK-based case study. NUFS. 2010;40:557-65.

8. Geaney F, Kelly C, Scotto Di Marrazzo J, Gilgan L, McCarthy M, Perry IJ. Evaluation of the uptake of voluntary calorie posting on menus in Ireland. Dublin: Department of Health, 2015.

9. Mah CL, Vanderlinden L, Mamatis D, Ansara DL, Levy J, Swimmer L. Ready for policy? Stakeholder attitudes toward menu labelling in Toronto, Canada. Can J Public Health. 2013;104:e229-34.

10. Almanza BA, Nelson D, Chai S. Obstacles to nutrition labeling in restaurants. J Am Diet Assoc. 1997;97:157-61.

11. Food Safety Authority of Ireland (FSAI). Calories on menus in Ireland. A report on a national consultation. Dublin: FSAI; 2012.

12. Jeong JY, Kim E, Yang IS, Ham S. Motivators and Barriers to Provision of Nutritional Information in Restaurants. Korean Journal of Hospitality & Tourism 2015;24:227-43.

13. Logue D, Kennelly J, Keaveney E, O’Connor D, Bhriain SN, Flynn M. Calorie menu labelling in Ireland: a survey of food service businesses. Proc Nutr Soc. 2013;72.

14. Maestro V, Salay E. Restaurant nutrition and health information in the municipality of Campinas, São Paulo, Brazil: expectations of managers with respect to benefits and obstacles. Journal of Foodservice. 2008;19:262-9.

15. Ottawa Public Health. Report on Ottawa Restaurant Survey. Ottawa: Ottawa Public Health; 2013.

16. Shupe E. Obstacles to Participation in Menu Labeling Observed by the Independent Foodservice Establishments [Degree of Doctor of Philosophy]. Minnesota, United States: Walden University; 2013.

17. Vyth EL, Van Der Meer EW, Seidell JC, Steenhuis IH. A nutrition labeling intervention in worksite cafeterias: an implementation evaluation across two large catering companies in the Netherlands. Health Promotion Int. 2011;27:230-7.
