# Supplementary material for: Barriers and facilitators to implementation of menu labelling interventions from a food service industry perspective: a mixed methods systematic review
Source: Int J Behav Nutr Phys Act. 2020 Apr 15;17:48. doi: 10.1186/s12966-020-00948-1 (PMC7161210; doi:10.1186/s12966-020-00948-1)
Supplement: Supplementary file 7 — Additional file 7. List of constructs and sample quotes following the deductive and inductive coding. This file provides results from the deductive and inductive coding, including themes under each construct and illustrative quotes. [file 12966_2020_948_MOESM7_ESM.docx]

| **Additional file 7** List of constructs and sample quotes following the deductive and inductive coding | | | |
| --- | --- | --- | --- |
| **CFIR Domain** | **Facilitators of Implementation** | **Barriers to Implementation** | **Sample Quotes** |
| **Intervention Characteristics** |  |  |  |
| Intervention Source | Shaping policy development [[1-3](#_ENREF_1)] | No data | *The over-riding reason for companies deciding to take part in the scheme was that they wanted to be involved in its development. Although this was a voluntary initiative, there was a general feeling that there was the possibility in the future that displaying CI might become mandatory, and they felt that by being part of the scheme they could help to shape any such requirement.* [Author interpreted summary (Clegg et al 2009 [[1](#_ENREF_1)])] - Facilitator |
| Evidence Strength & Quality | Evidence of effectiveness [[4](#_ENREF_4)]  A strong business case [[5](#_ENREF_5)] | Ambiguity around evidence of effectiveness [[4](#_ENREF_4), [6](#_ENREF_6), [7](#_ENREF_7)] | *I would look at other jurisdictions that have tried this approach and see if it is achieving the desired goals. If it is then I would take the best practices and implement.* [Quote from foodservice business participant (Ottawa Public Health 2013 [[4](#_ENREF_4)])] - Facilitator  *Several interviewees called into question the evidence on effectiveness of menu-labelling interventions to shape consumer behaviour* [Author interpreted summary (Mah et al 2013[[6](#_ENREF_6)])] - Barrier |
| Relative Advantage | Perceived benefits of menu labelling for participating businesses [[5](#_ENREF_5), [8](#_ENREF_8), [9](#_ENREF_9)]  Improved business image/reputation [[1-3](#_ENREF_1), [5](#_ENREF_5), [10-12](#_ENREF_10)]  Attracting/retaining customers [[1](#_ENREF_1), [3](#_ENREF_3), [6](#_ENREF_6), [7](#_ENREF_7), [10](#_ENREF_10)]  Increased sales/profitability [[11](#_ENREF_11), [13](#_ENREF_13)]  Cost savings [[2](#_ENREF_2), [5](#_ENREF_5)]  Increased customer trust/confidence [[3](#_ENREF_3), [11](#_ENREF_11)]  Opportunity for better customer service [[2](#_ENREF_2), [4](#_ENREF_4)]  Opportunity for creativity/learning [[2](#_ENREF_2), [14](#_ENREF_14)]  Menu labelling as a marketing tool [[15](#_ENREF_15)]  Building relationships with health authorities [[1](#_ENREF_1)] | Reduced sales/profitability [[1](#_ENREF_1), [4-6](#_ENREF_4), [9](#_ENREF_9), [12](#_ENREF_12), [13](#_ENREF_13), [15](#_ENREF_15), [16](#_ENREF_16)]  Loss of flexibility/creativity [[3](#_ENREF_3), [13](#_ENREF_13), [15](#_ENREF_15), [16](#_ENREF_16)]  Customer loss [[1](#_ENREF_1), [3](#_ENREF_3), [7](#_ENREF_7)]  Negative impact on image/brand [[3](#_ENREF_3)]  Lack of economic return on time investment [[12](#_ENREF_12)] | *Participants displaying calorie information had a strong desire to portray a positive company image to consumers. This desire served as a motivating factor for ensuring successful implementation. Such participants believed that displaying calories would help them to portray their businesses as dynamic and forward-thinking and thus they were supportive of implementation and were prepared to overcome perceived barriers* [Author interpreted summary (Geaney et al 2015[[3](#_ENREF_3)]) - Facilitator  *There was a concern amongst the participating companies that displaying CI on their products could potentially reduce sales or lose customers, if customers perceived the products to be too high in calories.* [Author interpreted summary (Clegg et al 2009 [[1](#_ENREF_1)])] - Barrier |
| Adaptability | No data | No data |  |
| Trialability | No data | No data |  |
| Complexity | No data | Implementation a time consuming process [[1-5](#_ENREF_1), [7](#_ENREF_7), [15](#_ENREF_15), [16](#_ENREF_16)]  Greater accuracy/precision in all cooking processes required [[5](#_ENREF_5), [15](#_ENREF_15)] | *Barriers perceived by restaurant staff… Standardised menus require more accuracy in all cooking processes* [Author interpreted summary (Zick et al 2010 [[15](#_ENREF_15)])] - Barrier  *The most common reasons reported by food service businesses for not displaying calorie information were that it was too time consuming to implement (32.6% n=183)* [Statistical data (Geaney et al 2015 [[3](#_ENREF_3)])] - Barrier |
| Design Quality & Packaging | Rigorous menu labelling format [[5](#_ENREF_5)]  Easy-to-understand scheme for businesses [[5](#_ENREF_5)]  Free advertising and nutritional analysis [[12](#_ENREF_12)]  Menu label stickers [[5](#_ENREF_5)]  Strong branding [[5](#_ENREF_5)] | Excess information on menus [[1](#_ENREF_1), [9](#_ENREF_9)]  Materials not tailored to fit space [[1](#_ENREF_1)]  Excess point-of-sale display options [[7](#_ENREF_7)]  Lack of customer education component [[3](#_ENREF_3)]  Insufficient nutrition information [[3](#_ENREF_3)]  Non user-friendly scheme – numerical format [[3](#_ENREF_3)] | *One health service trust manager also commented that part of the appeal of Caloriewise was that it was clearer and more rigorous than the ‘traffic light’ system (‘red-amber-green’ labelling to denote healthiness) that they had used previously.* [Author interpreted summary (Ray et al 2013 [[5](#_ENREF_5)])] - Facilitator  *For consistency I feel principle 2 has too many options and may not promote clarity.* [Quote from foodservice business participant (FSAI 2012 [[7](#_ENREF_7)])] - Barrier |
| Cost | Cost neutral [[3](#_ENREF_3)] | General cost of menu labelling [[5](#_ENREF_5), [10](#_ENREF_10), [17](#_ENREF_17)]  Nutritional analysis [[1](#_ENREF_1), [4](#_ENREF_4), [7](#_ENREF_7), [9](#_ENREF_9), [11](#_ENREF_11), [13](#_ENREF_13), [16](#_ENREF_16)]  Changing menus/displays [[1](#_ENREF_1), [3](#_ENREF_3), [4](#_ENREF_4), [6](#_ENREF_6), [9](#_ENREF_9)]  Printing nutrition information [[3](#_ENREF_3), [11](#_ENREF_11), [13](#_ENREF_13), [16](#_ENREF_16)]  Hiring consultants/appointing advisors [[3](#_ENREF_3), [7](#_ENREF_7), [9](#_ENREF_9)]  Purchasing nutrition analysis software [[3](#_ENREF_3), [7](#_ENREF_7)]  Staff time in implementation [[3](#_ENREF_3), [4](#_ENREF_4)]  Training staff [[7](#_ENREF_7)] | *For the businesses who had not rolled out the scheme in all their outlets there were concerns that to do so would lead to considerable costs – particularly in changing displays and menus outside of their normal cycle of change.* [Author interpreted summary (Clegg et al 2009 [[1](#_ENREF_1)])] – Barrier  *It is not as easy as saying. Definitely there will be lots of costs incurred on our part. Before implementing, we have to look in-depth on various factors. We need to change the menu with the elaborated ones. Every nutritional information to be displayed requires laboratory testing or analyses; advisor needs to be appointed as well as other requirements. All of these will directly creating unreasonable cost burden to the restaurant operators* [Quote from restaurant operator (Din et al 2012 [[9](#_ENREF_9)])] - Barrier |
| **Outer Setting** |  |  |  |
| Consumer Needs & Resources | Customer demand for/interest in menu labelling [[2-7](#_ENREF_2), [9](#_ENREF_9), [12](#_ENREF_12), [17](#_ENREF_17)]  Providing nutrition information to customers [[2](#_ENREF_2), [9](#_ENREF_9), [11](#_ENREF_11), [14](#_ENREF_14), [17](#_ENREF_17)]  Enabling informed food choices [[1](#_ENREF_1), [3](#_ENREF_3), [4](#_ENREF_4), [11](#_ENREF_11)]  Providing a consistent menu labelling scheme for customers [[5](#_ENREF_5)]  Improving customer health [[5](#_ENREF_5)] | Lack of customer demand for/interest in menu labelling [[3](#_ENREF_3), [4](#_ENREF_4), [6](#_ENREF_6), [9-11](#_ENREF_9), [14](#_ENREF_14), [16](#_ENREF_16)]  Risk of overwhelmed/confused customers [[1](#_ENREF_1), [3](#_ENREF_3), [5](#_ENREF_5), [7](#_ENREF_7), [15](#_ENREF_15)]  Interference with dining experience [[3](#_ENREF_3), [12](#_ENREF_12)] | *Looking at the increasing customers demand, yes, I have no objection and willing to make it available in the menu* [Quote from restaurant operator (Din et al 2012 [[9](#_ENREF_9)])] - Facilitator  *An important motivation for businesses’ involvement in the scheme was their commitment to enabling their customers to make informed choices about their food purchases* [Author interpreted summary (Clegg et al 2009 [[1](#_ENREF_1)])] – Facilitator  *When you come here for afternoon tea, it’s a treat, you know, it’s a real treat. You’re going to come, you’re going to have your lovely tea, you’re going to have a glass of champagne and the lovely pastries and scones and so on and so forth so do you really want to know about calories?* [Quote from head chef in independent hotel restaurant (Geaney et al 2015 [[3](#_ENREF_3)])] - Barrier |
| Cosmopolitanism | Support from existing contacts (external to food establishment) [[3](#_ENREF_3)]  Trust in/respect for health authorities [[3](#_ENREF_3)] | No data | *The use of existing contacts (for example, availing of services provided by a third party company already in use by the food service business) was highlighted as being particularly beneficial among all establishments displaying calories.* [Author interpreted summary (Geaney et al 2015 [[3](#_ENREF_3)])] - Facilitator |
| Peer Pressure | Gaining a competitive edge over other food establishments [[1-3](#_ENREF_1), [5](#_ENREF_5), [6](#_ENREF_6), [11](#_ENREF_11), [12](#_ENREF_12), [14](#_ENREF_14)]  Pressure to keep up with competing food establishments [[1](#_ENREF_1)] | Risk of losing market share to dishonest competitors [[3](#_ENREF_3)] | *For restaurants, the menu labelling program presented an opportunity to get a head start on their competition* [Author interpreted summary (Toronto Public Health 2015 [[2](#_ENREF_2)])] - Facilitator  *It was believed that such establishments would have the resources to employ “clever marketing tools” to conceal high calorie contents resulting in misleading information being presented to consumers. Single outlet establishment owners anticipated that they would be unable to compete in this instance due to limited resources* [Author interpreted summary (Geaney et al 2015 [[3](#_ENREF_3)])] - Barrier |
| Media & Societal Pressure* | Forced in the media [[11](#_ENREF_11)]  Family and friend influences [[12](#_ENREF_12)] | No data | *Subsequent key informant interview results indicated that motivating factors for participating restaurants included…..friend and family influences* [Author interpreted summary (Britt et al 2011 [[12](#_ENREF_12)])] - Facilitator |
| External Policy & Incentives | Anticipated future menu labelling policy/legislation [[1-5](#_ENREF_1), [12](#_ENREF_12)]  Introduced on mandatory basis [[3](#_ENREF_3), [4](#_ENREF_4), [8](#_ENREF_8), [9](#_ENREF_9), [11](#_ENREF_11)]  Monitoring and enforcement [[3](#_ENREF_3), [5](#_ENREF_5), [9](#_ENREF_9)]  Financial support from government [[3](#_ENREF_3), [9](#_ENREF_9)]  Voluntary menu labelling scheme in situ [[3](#_ENREF_3)]  Ability to champion a voluntary approach [[5](#_ENREF_5)]  Participation in governmental policies [[11](#_ENREF_11)] | Absence of legislation requiring mandatory provision [[3-6](#_ENREF_3), [9](#_ENREF_9), [10](#_ENREF_10), [16](#_ENREF_16)]  Lack of monitoring and enforcement [[3](#_ENREF_3), [9](#_ENREF_9), [11](#_ENREF_11)]  Lack of guidelines [[11](#_ENREF_11), [13](#_ENREF_13), [15](#_ENREF_15)].  Fears of liability due to inaccurate information [[1](#_ENREF_1), [4](#_ENREF_4), [5](#_ENREF_5)]  Excessive bureaucratic burdens on businesses [[4](#_ENREF_4), [7](#_ENREF_7), [16](#_ENREF_16)] | *If it is not mandatory and there is no enforcement, who bothers to do so? I mean, voluntary does not work.* [Quote from restaurant operator (Din et al 2012 [[9](#_ENREF_9)])] - Barrier  *Barriers to participation in the scheme for some food businesses were….. fears of liability if calorie information was found to be inaccurate.* [Author interpreted summary (Ray et al 2013 [[5](#_ENREF_5)])] - Barrier  *While there was a degree of apprehension surrounding the logistics of policing the proposed legislation, there was general agreement that rigorous enforcement and regulation by health authorities would be a key potential facilitating factor in ensuring that calorie posting is applied in a standardised way.* [Author interpreted summary (Geaney et al 2015 [[3](#_ENREF_3)])] - Facilitator |
| Economic Climate* | No data | Poor economic conditions [[4](#_ENREF_4), [5](#_ENREF_5), [7](#_ENREF_7)] | *The current economic climate was also felt to be an additional disincentive to participation: ‘… at this time in the financial cycle, I’m sure a lot of restaurants out there will go … ‘We need bums on seats and is the Caloriewise scheme going to work, is that going to do it for us?’ So that’s the hard-nosed financial reality in restaurants at the moment.’* [Author interpreted summary and quote from restaurant head office (Ray et al 2013 [[5](#_ENREF_5)])] - Barrier |
| Educational System* | Nutrition education in catering school curriculum [[5](#_ENREF_5)]  Education on healthy eating from early age [[3](#_ENREF_3)] | No data | *In the longer term, respondents thought that training on calorie and nutritional information should be introduced as part of basic food hygiene training (a mandatory requirement for catering staff) and that more emphasis on training and awareness raising of healthy eating and nutrition among chefs, catering managers and others in the hospitality industry was needed.* [Author interpreted summary (Ray et al 2013 [[5](#_ENREF_5)])] – Facilitator |
| **Inner Setting** |  |  |  |
| Structural Characteristics | Part of a franchise/chain [[3](#_ENREF_3), [7](#_ENREF_7)]  Larger food businesses [[5](#_ENREF_5)]  Smaller food businesses [[6](#_ENREF_6)]  Central coordination [[14](#_ENREF_14)] | Smaller food businesses [[2](#_ENREF_2), [4](#_ENREF_4), [5](#_ENREF_5), [7](#_ENREF_7), [10](#_ENREF_10)]  Independent restaurants [[2](#_ENREF_2), [4](#_ENREF_4), [5](#_ENREF_5), [7](#_ENREF_7)]  Franchise/chain businesses [[4](#_ENREF_4), [5](#_ENREF_5)]  Fine dining restaurants [[3](#_ENREF_3), [4](#_ENREF_4)] | *As a small, independent restaurant that changes our menu often, it would be prohibitively expensive for us to produce nutritional labelling.* [Quote from foodservice business participant (Ottawa Public Health 2013 [[4](#_ENREF_4)])] - Barrier  *I suppose for us, we don’t have to go looking for all this information you know, it’s there for us; we get emails, we get all the POS (point of sale) material for in-store so it’s made quite simple for a franchisee here.* [Quote from fast food franchise (Geaney et al 2015 [[3](#_ENREF_3)])] - Facilitator  *The same small chain suggested that smaller companies, in contrast to large ones, could more readily and feasibly adapt menus to provide nutrition information, since they were less embedded in complex supply chains.* [Author interpreted summary (Mah et al 2013 [[6](#_ENREF_6)])] - Facilitator |
| Networks & Communications | Internal communication [[15](#_ENREF_15)] | No data | *Communication between different areas of the hotel was also vital for success. Chefs needed to become familiar cooking with weighing scales and precise recipes, conversing with the nutritionist and ensuring the administration team had the correct nutrition information to present on the menu.* [Author interpreted summary (Zick et al 2010 [[15](#_ENREF_15)])] - Facilitator |
| Culture | No data | No data |  |
| Implementation Climate |  |  |  |
| 1. Tension for change | No data | Belief that menu labelling is unnecessary [[3](#_ENREF_3), [4](#_ENREF_4), [7](#_ENREF_7), [9](#_ENREF_9), [16](#_ENREF_16)] | *We don’t believe that this information is necessary in a restaurant as people can get this information through various other methods.* [Quote from foodservice business participant (FSAI 2012 [[7](#_ENREF_7)])] - Barrier |
| 1. Compatibility | Alignment with existing practices/initiatives [[1](#_ENREF_1), [2](#_ENREF_2), [5](#_ENREF_5), [11](#_ENREF_11), [13](#_ENREF_13), [16](#_ENREF_16)]  Sense of responsibility [[5](#_ENREF_5), [6](#_ENREF_6), [11](#_ENREF_11), [13](#_ENREF_13), [16](#_ENREF_16)]  Standardised recipes/menus [[3-5](#_ENREF_3)]  Alignment with business values/ethos [[5](#_ENREF_5)]  Adaptable menus [[6](#_ENREF_6)]  Using own-brand products [[5](#_ENREF_5)] | Lack of standardised recipes/menus [[1-5](#_ENREF_1), [7](#_ENREF_7), [10](#_ENREF_10), [11](#_ENREF_11), [16](#_ENREF_16)]  Limited space on menu [[1](#_ENREF_1), [3-5](#_ENREF_3), [7](#_ENREF_7), [11](#_ENREF_11), [13](#_ENREF_13), [15](#_ENREF_15), [16](#_ENREF_16)]  Frequent menu changes/variations [[1](#_ENREF_1), [3-7](#_ENREF_3), [13](#_ENREF_13)]  Too many products/menu items [[1](#_ENREF_1), [4](#_ENREF_4), [5](#_ENREF_5), [7](#_ENREF_7), [16](#_ENREF_16)]  No sense of responsibility [[4](#_ENREF_4), [7](#_ENREF_7), [16](#_ENREF_16)]  Lack of alignment with food served/aesthetics [[3](#_ENREF_3), [4](#_ENREF_4), [16](#_ENREF_16)]  Fast-pace environment [[3](#_ENREF_3)]  Lack of mass-produced food [[7](#_ENREF_7)] | *Logistical concerns were raised over the lack of menu space to display calories* [Author interpreted summary (Geaney et al 2015 [[3](#_ENREF_3)])] - Barrier  *Other companies, however, cited difficulties standardising freshly made-to order products, like a pizza: ‘It’s challenging also because we’re a handmade product […], you know, the cheese is sprinkled on by hand, okay the number of toppings […] would be set, but you scatter by hand, so it’s very difficult to always guarantee the nutritional value of each pizza.’* [Author interpreted summary and quote from fast food head office (Ray et al 2013 [[5](#_ENREF_5)])] - Barrier  *Some respondents – particularly those in the health service trusts – believed that their organisation had a responsibility to try to improve public health. They were already involved in healthy-eating schemes and saw Caloriewise as a step towards tackling obesity issues* [Author interpreted summary (Ray et al 2013 [[5](#_ENREF_5)])] - Facilitator |
| 1. Relative Priority | No data | Competing demands [[2-4](#_ENREF_2), [6](#_ENREF_6), [16](#_ENREF_16)]  Not a priority [[4](#_ENREF_4), [5](#_ENREF_5)] | *64% felt that they were too busy to “figure out” nutrition information provision* [Statistical data (Mah et al 2013 [[6](#_ENREF_6)])] - Barrier  *Would take too much energy and time. My efforts can go into providing for my guests.* [Quote from foodservice business participant (Ottawa Public Health, 2013 [[4](#_ENREF_4)])] - Barrier |
| 1. Incentives & Rewards | No data | No financial incentive [[4](#_ENREF_4)] | *Extra work and not getting paid for it.* [Quote from foodservice business participant (Ottawa Public Health, 2013 [[4](#_ENREF_4)])] - Barrier |
| 1. Goals & Feedback | Supports business profitability goals [[3](#_ENREF_3), [11](#_ENREF_11), [13](#_ENREF_13)] | Impedes business profitability goals [[1](#_ENREF_1), [3](#_ENREF_3), [4](#_ENREF_4), [6](#_ENREF_6), [9](#_ENREF_9), [12](#_ENREF_12), [13](#_ENREF_13), [15](#_ENREF_15), [16](#_ENREF_16)] | *You know, the bottom line of any business besides satisfying customer is making most profit out of it. Operators will not do anything that might decrease their business profit or the customer’s purchasing.* [Quote from restaurant operator (Din et al 2012 [[9](#_ENREF_9)])] - Barrier  *For foodservices not currently using nutrition labels, the research and development directors' perceptions about the effect of nutrition labeling on sales helped explain their willingness to use labeling. The regression model had an F value of 5.046 and a P value of .0092. The difference in willingness between those who thought nutrition labeling would improve sales and those who thought it would decrease sales was 3.9 points on a 10-point scale. Those directors who thought nutrition labeling would increase sales were more willing to implement labeling than those who thought it would have no effect…*[Statistical data (Almanza et al 1997 [[13](#_ENREF_13)])] - Facilitator |
| 1. Learning Climate | No data | No data |  |
| Readiness for Implementation |  |  |  |
| 1. Leadership Support | Head office supporting outlet teams [[3](#_ENREF_3), [4](#_ENREF_4)] | No data | *The head office would have to supply the information.* [Quote from foodservice business participant (Ottawa Public Health 2013 [[4](#_ENREF_4)])] - Facilitator |
| 1. Available Resources | External:  Access to financial support [[4](#_ENREF_4), [5](#_ENREF_5), [7](#_ENREF_7)] | Internal:  Lack of time [[1-5](#_ENREF_1), [7](#_ENREF_7), [11](#_ENREF_11), [13](#_ENREF_13), [16](#_ENREF_16)]  Lack of money [[3-5](#_ENREF_3), [9](#_ENREF_9)]  Limited staff [[2](#_ENREF_2), [4](#_ENREF_4), [5](#_ENREF_5), [16](#_ENREF_16)]  Short-time building lease [[4](#_ENREF_4)] | *For a future rollout, business respondents thought it important for the FSA to provide…. some financial support for small businesses for the production of point of sale materials, advertising and scheme promotion.* [Author interpreted summary (Ray et al 2013 [[5](#_ENREF_5)])] - Facilitator  *I would have to calculate the calories every single day and I have not got time for that, basically.* [Quote from owner/head chef of independent restaurant (Geaney et al 2015 [[3](#_ENREF_3)])] - Barrier |
| 1. Access to Knowledge & Information | Internal:  Staff training and support [[5](#_ENREF_5), [14](#_ENREF_14), [15](#_ENREF_15)]  Access to information [[3-5](#_ENREF_3)]  Access to (user-friendly) nutrition analysis software [[3](#_ENREF_3), [5](#_ENREF_5)]  Access to in-house technician [[10](#_ENREF_10)]  External:  Access to knowledge and information from designers/professionals/health agency/suppliers [[1-5](#_ENREF_1), [7](#_ENREF_7), [12](#_ENREF_12)]  Access to user-friendly (approved) nutrition analysis software from health agency [[3](#_ENREF_3), [5](#_ENREF_5), [7](#_ENREF_7)]  Access to training from health agency/professionals [[3](#_ENREF_3), [5](#_ENREF_5)] | Internal:  Lack of nutrition expertise [[3-5](#_ENREF_3), [10](#_ENREF_10), [11](#_ENREF_11), [13](#_ENREF_13), [17](#_ENREF_17)]  Challenges acquiring nutrition information [[2](#_ENREF_2), [4](#_ENREF_4), [5](#_ENREF_5)]  Lack of reliable nutrition information [[5](#_ENREF_5), [10](#_ENREF_10)]  Lack of training and support [[3](#_ENREF_3), [5](#_ENREF_5)]  Lack of nutrition analysis software [[11](#_ENREF_11)]  Difficulties with business systems [[1](#_ENREF_1)]  External:  Difficulty obtaining information from suppliers/health agency [[1](#_ENREF_1), [5](#_ENREF_5), [13](#_ENREF_13), [16](#_ENREF_16)]  Non user-friendly nutrition analysis software [[2](#_ENREF_2), [3](#_ENREF_3)]  Discrepancy in nutrition information obtained from suppliers [[3](#_ENREF_3)]  Excessive information from health agency [[5](#_ENREF_5)] | *Obtaining information from suppliers could take more time than business had anticipated, which in turn led to delays in providing calorie information in some outlets.* [Author interpreted summary (Clegg et al 2009 [[1](#_ENREF_1)])] - Barrier  *Regarding the level of importance of reasons not to provide nutrition information in relation to the type of restaurants, fast food managers reported a higher importance for the factor ‘Little knowledge on the subject’ (50%, at levels very and very much important)* [Statistical data (Maestro et al 2008 [[10](#_ENREF_10)])] - Barrier  *Another participant felt that it would have been useful to have ‘a Caloriewise guru' to provide bespoke on-site advice on displaying calories.* [Author interpreted summary (Ray et al 2013 [[5](#_ENREF_5)])] - Facilitator  *This company was more easily able to expand the scheme because it had developed calorie counting software in-house and had already input information for all the items in its recipe bank.* [Author interpreted summary (Ray et al 2013 [[5](#_ENREF_5)])] - Facilitator  *Regular nutrition training for employees to enable them to advise customers on menu choices was considered a necessity.* [Author interpreted summary (Zick et al 2010 [[15](#_ENREF_15)])] - Facilitator |
| **Characteristics of Individuals** |  |  |  |
| Knowledge & Beliefs about Menu Labelling | Positive view of scheme [[3](#_ENREF_3), [5](#_ENREF_5), [12](#_ENREF_12)]  Belief that right thing to do [[3](#_ENREF_3)] | Belief that it is impractical and burdensome [[2](#_ENREF_2), [4](#_ENREF_4), [7](#_ENREF_7), [16](#_ENREF_16)]  Aversion to menu labelling [[7](#_ENREF_7), [13](#_ENREF_13), [17](#_ENREF_17)] | *This will be impossible to quantify and far too difficult to monitor.* [Quote from foodservice business participant (FSAI 2012 [[7](#_ENREF_7)])] - Barrier  *Again this is entirely unworkable in restaurants. Chefs are humans, not robots.* [Quote from foodservice business participant (FSAI 2012 [[7](#_ENREF_7)])] - Barrier  *Subsequent key informant interview results indicated that motivating factors for participating restaurants included…..positive view of menu labelling* [Author interpreted summary (Britt et al 2011 [[12](#_ENREF_12)])] - Facilitator  *The majority of businesses displaying calories felt it was the right thing to do (62%, n=26)* [Statistical data (Geaney et al 2015 [[3](#_ENREF_3)])] - Facilitator |
| Self-efficacy | No data | No data |  |
| Individual Stage of Change | No data | No data |  |
| Individual Identification with the Food Business | No data | No data |  |
| Other Personal Attributes | Leadership experience [[16](#_ENREF_16)] | Apathy towards menu labelling [[2](#_ENREF_2), [16](#_ENREF_16)] | *The restaurant simply did not have the…. motivation to do it.* [Author interpreted summary [Toronto Public Health 2015 [[2](#_ENREF_2)])] - Barrier  *The years of leadership experience was the independent variable that was compared to the dependent variable of willingness to participate with menu labeling. As demonstrated in table 8, …. the statistics were able to reject the null hypothesis, indicating that there was a statistical significance relationship between years of leadership experience and willingness to participate with menu labeling.* [Statistical data (Shupe 2013 [[16](#_ENREF_16)])] - Facilitator |
| **Process** |  |  |  |
| Planning | No data | No data |  |
| Engaging | Outreach support for smaller businesses [[5](#_ENREF_5)] | No data | *Outreach support for smaller businesses to encourage them to take the process on board was also thought necessary.* [Author interpreted summary [Ray et al 2013 [[5](#_ENREF_5)])] - Facilitator |
| 1. Opinion Leaders | Advice from other businesses with experience [[1](#_ENREF_1)] | No data | *Companies who had branches and/or headquarters in the USA asked their US counterparts for advice about displaying the CI, as there are areas of the USA, for example New York City, where this is now required by law, therefore they had been through a similar process in the recent past.* [Author interpreted summary [Clegg et al 2013 [[1](#_ENREF_1)])] - Facilitator |
| 1. Formally Appointed Internal Implementation Leaders | No data | No data |  |
| 1. Champions | No data | No data |  |
| 1. External Change Agents | Health agency/professionals providing access to knowledge and information [[1-5](#_ENREF_1), [7](#_ENREF_7), [12](#_ENREF_12)]  Health agency providing user-friendly (approved) nutrition analysis software [[3](#_ENREF_3), [5](#_ENREF_5), [7](#_ENREF_7)]  Health agency/professionals providing access to training from health agency/professionals [[3](#_ENREF_3), [5](#_ENREF_5)]  Health agency providing access to financial support  [[4](#_ENREF_4), [5](#_ENREF_5), [7](#_ENREF_7)] | Health agency providing access to non-user-friendly nutrition software [[2](#_ENREF_2), [3](#_ENREF_3)]  Health agency providing insufficient information [[5](#_ENREF_5)]  Health agency providing excess information [[5](#_ENREF_5)] | *There were examples of companies who, whilst not changing their displays greatly, did employ designers to help them to decide where and how the CI should appear.* [Author interpreted summary [Clegg et al 2013 [[1](#_ENREF_1)])] - Facilitator  *Food service businesses reported ‘training and advice from professionals’ as the main factor that would encourage them to implement and display calorie information (37.9%, n=213).* [Statistical data (Geaney et al 2015 [[3](#_ENREF_3)])] - Facilitator  *When I kind of first looked at it I thought this is going to be pretty easy, but it wasn’t. I needed to be a lot more specific - that’s why when the two researchers came out and photographed our larder and our fridge, you know, it proved a lot easier, because they then knew what I was talking about.* [Quote from restaurant head office (Ray et al 2013 [[5](#_ENREF_5)])] - Facilitator  *Another respondent said that the information from the FSA was detailed but quite confusing for people who had never been involved in calorie labelling before*…[Author interpreted summary (Ray et al 2013 [[5](#_ENREF_5)])] - Barrier |
| 1. Internal Key Stakeholders* | Head office working closely with outlet staff [[1](#_ENREF_1)] | Difficulty in training employees [[13](#_ENREF_13), [16](#_ENREF_16)]  Lack of compliance with protocols [[3](#_ENREF_3), [5](#_ENREF_5)]  Perceived threat to chef’s authority [[4](#_ENREF_4)]  Head office not working closely with outlet staff [[1](#_ENREF_1)]  Lack of internal buy-in [[5](#_ENREF_5)] | *Where there had been close liaison the outlet managers knew what the scheme was about, knew what the materials would be like before they arrived, and had sometimes been instrumental in deciding where they would be displayed in their outlet. In these cases the outlet staff felt engaged with the scheme and felt a part of it.* [Author interpreted summary (Clegg et al 2009 [[1](#_ENREF_1)])] - Facilitator  *For operations currently providing nutrition labelling only one obstacle was significant at α=0.05: “difficulty in training employees how to implement nutrition labelling”.*  [Statistical data (Almanza et al 1997 [[13](#_ENREF_13)])] - Barrier  *An on-going challenge, particularly for chain businesses, was raised regarding franchisee and individual retailers’ lack of compliance with measurement protocols; resulting in inaccurate calorie information being presented to customers.* [Author interpreted summary (Geaney et al 2015 [[3](#_ENREF_3)])] - Barrier |
| 1. External Key Stakeholders* | Engaging suppliers [[5](#_ENREF_5), [7](#_ENREF_7)]  Access to nutrition information provided by suppliers [[5](#_ENREF_5)] | Lack of information provided by purveyors/suppliers [[1](#_ENREF_1), [5](#_ENREF_5), [13](#_ENREF_13), [16](#_ENREF_16)]  Discrepancy in nutrition information provided by suppliers [[3](#_ENREF_3)]  Inconsistency in ingredients provided by suppliers [[2](#_ENREF_2), [4](#_ENREF_4)]  Lack of standardized products from purveyors [[13](#_ENREF_13), [16](#_ENREF_16)] | *Obtaining information from suppliers could take more time than business had anticipated, which in turn led to delays in providing calorie information in some outlets.*  [Author interpreted summary (Clegg et al 2009 [[1](#_ENREF_1)])] - Barrier  *It will need support from the suppliers in order to ensure you have as complete a database as possible … if rolling this out further, it would require a more targeted approach in terms of getting all the catering suppliers on board, providing the details of their products.* [Quote from foodservice business participant (Ray et al 2013 [[5](#_ENREF_5)])] - Facilitator |
| Executing | No data | Inability to provide accurate nutrition information [[3](#_ENREF_3), [4](#_ENREF_4), [11](#_ENREF_11)] | *Concerns regarding the inability to provide accurate calorie information was highlighted as both an anticipated barrier (from the perspective of those not displaying calories) and a realised barrier (by those currently displaying calories).* [Author interpreted summary (Geaney et al 2015 [[3](#_ENREF_3)])] - Barrier |
| Reflecting & Evaluating | Measuring, observing and communicating results [[8](#_ENREF_8)] | No data | *For a high promotion implementation…result demonstrability (OR ¼ 1.51, 95% CI:1.07–2.13, p ¼ 0.021) were the best predictors.* [Statistical data (Vyth et al 11 [[8](#_ENREF_8)])] - Facilitator |
| Adapting the Organisation* | Adapting menus to lower calorie content [[3](#_ENREF_3), [15](#_ENREF_15)]  Utensils to assist with standardising recipes [[3](#_ENREF_3), [5](#_ENREF_5)]  Enforcing stricter adherence to recipes [[5](#_ENREF_5)]  Reducing product/menu item range [[5](#_ENREF_5)]  Adding extra requirements in supplier contracts [[5](#_ENREF_5)] | No data | *One of the things that we did do was we empowered a lot of our franchisees with tools to make sure the quantity of product that were going on the pizza were consistent. So we developed a cup system where there are individual measurements inside the cup. So for a 7 inch pizza, you would go to the 1st line and for a 9 inch you would go to the second line….So it was getting that consistency and giving the tools to the franchisees to make that happen.* [Quote from managing director of fast food chain (Geaney et al 2015 [[3](#_ENREF_3)])] - Facilitator  *One catering outlet, in a hospital trust, had introduced the use of a standardised scoop for serving sandwich fillings specifically as a result of the Caloriewise scheme, in order to ensure that the calorie counts were accurate.* [Author interpreted summary (Ray et al 2013 [[5](#_ENREF_5)])] - Facilitator |
| Adapting the Intervention* | Actual adaptations  Providing nutrition information on select menu items only [[1](#_ENREF_1)]  Providing additional nutrition information alongside calories [[3](#_ENREF_3)]  Providing the average calorie content of menu items [[1](#_ENREF_1)]  Providing a caveat on the menu which states the possibility of some variation from stated calorific values [[1](#_ENREF_1)]  Incorporating a marketing element alongside menu labelling [[3](#_ENREF_3)]  Improving typographical presentation of nutrition information [[15](#_ENREF_15)]  Suggested adaptations  Different display options available to fit space [[1](#_ENREF_1)]  Display only important nutritional information [[9](#_ENREF_9)]  Display additional information alongside nutrition (e.g. vegan, local) [[4](#_ENREF_4)]  Incorporate consumer education campaign alongside menu labelling [[3](#_ENREF_3)]  Provide a user-friendly menu labelling format (such as interpretive labelling) for customers [[3](#_ENREF_3), [4](#_ENREF_4)]  Provide an easy-to-understand scheme for businesses [[3](#_ENREF_3)]  Provide a separate brochure/file for nutrition information [[4](#_ENREF_4), [7](#_ENREF_7), [12](#_ENREF_12)]  Provide nutrition information available on request only [[4](#_ENREF_4), [16](#_ENREF_16)]  Limit point-of-sale display options [[7](#_ENREF_7)] | No data | *Companies wanted the CI to be user-friendly for their customers, therefore, they wanted their displays to give enough information, but not too much so that people were overwhelmed (this led to some companies deciding not to display CI for products where there were many options, such as coffees).* [Author interpreted summary (Clegg et al 2009 [[1](#_ENREF_1)])] - Facilitator  *The majority of establishments displaying calorie information displayed additional nutrition information alongside it. Among those displaying this information, it was felt that customers sought more than just calorie information alone.* [Author interpreted summary (Geaney et al 2015 [[3](#_ENREF_3)])] - Facilitator  *Nonparticipating restaurants made several suggestions about how the Heath Department could work with restaurants to overcome these barriers. Suggestions included the following: allow a separate brochure/menu for nutrition information…*[Author interpreted summary (Britt et al 2011 [[12](#_ENREF_12)])] - Facilitator |
| Trialing* | Pilot test before rolling out [[5](#_ENREF_5)] | No data | *All but one business decided to launch Caloriewise in certain outlets only. This was primarily because they wanted to test the scheme at a small number of sites before deciding whether to roll it out further.* [Author interpreted summary (Ray et al 2013 [[5](#_ENREF_5)])] - Facilitator |
| Scaling Up* | Gradually introducing labels across full menu [[5](#_ENREF_5)]  Working together to achieve smooth roll out [[1](#_ENREF_1)] | No data | *Displaying CI only on selected items allowed businesses to introduce CI gradually, as they often felt that to cover all dishes would be too much at once. This was particularly the case for those with very large and/or frequently rotating menus.* [Author interpreted summary (Ray et al 2013 [[5](#_ENREF_5)])] – Facilitator  *…head office staff should link up with outlet staff to ensure a smooth roll out…*[Author interpreted summary (Clegg et al 2009 [[1](#_ENREF_1)])] - Facilitator |

* new constructs generated inductively

**References**

1. Clegg S, Jordan E, Slade Z. An Evaluation of Provision of Calorie Information by Catering Outlets. United Kingdom: Food Standards Agency, 2009.

2. Toronto Public Health. Voluntary Menu Labelling Pilot Project: Final Report. Ontario, Canada: Toronto Public Health; 2015.

3. Geaney F, Kelly C, Scotto Di Marrazzo J, Gilgan L, McCarthy M, Perry IJ. Evaluation of the uptake of voluntary calorie posting on menus in Ireland. Dublin: Department of Health, 2015.

4. Ottawa Public Health. Report on Ottawa Restaurant Survey. Ottawa: Ottawa Public Health; 2013.

5. Ray K, Clegg S, Davidson R, Vegeris S. Evaluation of Caloriewise: A Northern Ireland pilot of the display of calorie information in food catering businesses. Northern Ireland: Food Standards Agency; 2013.

6. Mah CL, Vanderlinden L, Mamatis D, Ansara DL, Levy J, Swimmer L. Ready for policy? Stakeholder attitudes toward menu labelling in Toronto, Canada. Can J Public Health. 2013;104:e229-34.

7. Food Safety Authority of Ireland (FSAI). Calories on menus in Ireland. A report on a national consultation. Dublin: FSAI; 2012.

8. Vyth EL, Van Der Meer EW, Seidell JC, Steenhuis IH. A nutrition labeling intervention in worksite cafeterias: an implementation evaluation across two large catering companies in the Netherlands. Health Promotion Int. 2011;27:230-7.

9. Din N, Zahari MSM, Othman CN, Abas R. Restaurant operator's receptiveness towards providing nutritional information on menu. Procedia Soc Behav Sci 2012;50:699-709.

10. Maestro V, Salay E. Restaurant nutrition and health information in the municipality of Campinas, São Paulo, Brazil: expectations of managers with respect to benefits and obstacles. Journal of Foodservice. 2008;19:262-9.

11. Jeong JY, Kim E, Yang IS, Ham S. Motivators and Barriers to Provision of Nutritional Information in Restaurants. Korean Journal of Hospitality & Tourism 2015;24:227-43.

12. Britt JW, Frandsen K, Leng K, Evans D, Pulos E. Feasibility of voluntary menu labeling among locally owned restaurants. Health Promot Pract. 2011;12:18-24.

13. Almanza BA, Nelson D, Chai S. Obstacles to nutrition labeling in restaurants. J Am Diet Assoc. 1997;97:157-61.

14. Roodenburg AC, Payens IJ, Vrijhof C. Menu labeling in “out-of-home” sector: opportunities, barriers, and needs with respect to use of health communication in restaurants. Ann Nutr Metab. 2013;63(suppl1):1054.

15. Zick A, Wake Y, Reeves S. Nutrition labelling in restaurants: a UK-based case study. NUFS. 2010;40:557-65.

16. Shupe E. Obstacles to Participation in Menu Labeling Observed by the Independent Foodservice Establishments [Degree of Doctor of Philosophy]. Minnesota, United States: Walden University; 2013.

17. Logue D, Kennelly J, Keaveney E, O’Connor D, Bhriain SN, Flynn M. Calorie menu labelling in Ireland: a survey of food service businesses. Proc Nutr Soc. 2013;72.
