# Supplementary material for: Barriers and facilitators to implementation of menu labelling interventions from a food service industry perspective: a mixed methods systematic review
Source: Int J Behav Nutr Phys Act. 2020 Apr 15;17:48. doi: 10.1186/s12966-020-00948-1 (PMC7161210; doi:10.1186/s12966-020-00948-1)
Supplement: Supplementary file 8 — Additional file 8. Construct relationships. This file provides information on construct relationships and recommendations, including illustrative quotes. [file 12966_2020_948_MOESM8_ESM.docx]

| **Additional file 8** Construct relationships | | |
| --- | --- | --- |
| **Relationships** | **Sample Quotes** | **Papers** |
| Structural Characteristics (+) > Compatibility (+) | *Belonging to a franchise group and the use of standardised menus also facilitated the ease of implementation.* [Author interpreted summary (Geaney et al 2015 [[1](#_ENREF_1)])]  *The same small chain suggested that smaller companies, in contrast to large ones, could more readily and feasibly adapt menus to provide nutrition information, since they were less embedded in complex supply chains.* [Author interpreted summary (Mah et al 2013 [[2](#_ENREF_2)])] | [[1](#_ENREF_1), [2](#_ENREF_2)] |
| Structural Characteristics (-) > Compatibility (-) | *A lot of extra work. Out of scope of reality for a small restaurant. Our menu changes daily*. [Quote from foodservice business participant. (Ottawa Public Health 2013 [[3](#_ENREF_3)])]  *There are simply too many variables to make this work. Restaurants are not like chains – our food is not standard – our chefs can make changes to dishes at will. Chef A may make curry one way, while Chef B adds more cream because they are free-pouring instead of using a jug. The calorie content will change every time.* [Quote from foodservice business participant (FSAI 2012 [[4](#_ENREF_4)])] | [[3-5](#_ENREF_3)] |
| Structural Characteristics (+) > Compatibility (+) AND Access to Knowledge & Information (+) | *A few of the larger businesses were already using standardised, documented recipes across their sites, and so found this process much more straightforward. For example, one respondent described how all the recipes used in their outlets were produced centrally by their head chefs and stored on an online database. Outlets could select from a database of thousands of recipes. As a result, this company found that, although time consuming, calculating calories for menu items was a relatively easy task.* [Author interpreted summary (Ray et al 2013 [[5](#_ENREF_5)])]  *I suppose for us, we don’t have to go looking for all this information you know, it’s there for us; we get emails, we get all the POS (point of sale) material for in-store so it’s made quite simple for a franchisee here.* [Quote from franchise fast food chain (Geaney et al 2015 [[1](#_ENREF_1)])]. | [[1](#_ENREF_1), [5](#_ENREF_5)] |
| Structural Characteristics (-) > Compatibility (-) AND Cost (-) | *As a small, independent restaurant that changes our menu often, it would be prohibitively expensive for us to produce nutritional labelling.* *[Quote from foodservice business participant.* (Ottawa Public Health 2013 [[3](#_ENREF_3)])]  *It's a cost on small business, while fast food chains - who have standardized menus - can produce this once for all their outlets. Why punish independent restaurants?* [Quote from foodservice business participant (Ottawa Public Health 2013 [[3](#_ENREF_3)])] | [[3](#_ENREF_3)] |
| Structural Characteristics (-) > Available Resources (-) | *It would be a difficult task. I am a single owner and I don't have the time to do much about it.* [Quote from foodservice business participant (Ottawa Public Health 2013 [[3](#_ENREF_3)])] | [[3-6](#_ENREF_3)] |
| Structural Characteristics (-) > Available Resources (-) AND Access to Knowledge & Information (-) | *Barriers to participation in the scheme for some food businesses were identified as… resource constraints, particularly for smaller businesses with limited technical expertise.* [Author interpreted summary (Ray et al 2013 [[5](#_ENREF_5)])] | [[5](#_ENREF_5)] |
| Structural Characteristics (-) > Available Resources (-) > Engaging: External Change Agents (-) | *Single outlet owners and participants from small chain establishments not displaying calories, expressed their financial inability to outsource (e.g. hire an external nutritionist), as a prominent barrier to implementation.* [Author interpreted summary (Geaney et al 2015 [[1](#_ENREF_1)])] | [[1](#_ENREF_1), [4](#_ENREF_4)] |
| Structural Characteristics (+) > Available Resources (+) > Engaging: External Change Agents (+) | *Participants of large chain restaurants displaying calories identified their financial ability to outsource (i.e. hire external 3rd party company) as a key facilitating factor for implementation as this significantly eased workload and diminished accuracy concerns.* [Author interpreted summary (Geaney et al 2015 [[1](#_ENREF_1)])] | [[1](#_ENREF_1)] |
| Structural Characteristics (-) > Cost (-) | *The comparison in relation to the restaurant size was signiﬁcant only for the factor ‘Increase of the operational cost’, which the managers of microcompanies (47.2%, at levels very and very much important) considered to be more important than bigger companies (ANOVA; P = 0.048).* [Statistical data (Maestro et al 2008 [[7](#_ENREF_7)])] | [[3](#_ENREF_3), [7](#_ENREF_7)] |
| Structural Characteristics (-) AND Cost (-) > Goals & Feedback (-) > Relative Advantage (-) | *Adding extra cost in any form would run the small independent person out of business*. [Quote from foodservice business participant (Ottawa Public Health 2013 [[3](#_ENREF_3)])] | [[3](#_ENREF_3)] |
| Structural Characteristics (-) > Knowledge & Beliefs (-) | *Very small restaurant. It would be very difficult.* [Quote from foodservice business participant (Ottawa Public Health 2013 [[3](#_ENREF_3)])] | [[3](#_ENREF_3)] |
| Design Quality & Packaging (-) > Adapting the Intervention (+) | *A lot of things need to be considered before we can do that and not all nutrients or nutritional information can be displayed but those important ones only*. [Quote from restaurant operator (Din et al 2012 [[8](#_ENREF_8)])] | [[4](#_ENREF_4), [8](#_ENREF_8)] |
| Goals & Feedback (-) > Relative Advantage (-) | *You know, the bottom line of any business besides satisfying customer is making most profit out of it. Operators will not do anything that might decrease their business profit or the customer’s purchasing power.* [Quote from restaurant operator (Din et al 2012 [[8](#_ENREF_8)])] | [[1-3](#_ENREF_1), [8-13](#_ENREF_8)] |
| Goals & Feedback (+) > Relative Advantage (+) | *Directors who thought nutrition labeling would increase sales were more willing to implement labeling than those who thought it would have no effect.* [Author interpreted summary (Almanza et al 1997 [[11](#_ENREF_11)])] | [[1](#_ENREF_1), [11](#_ENREF_11), [14](#_ENREF_14)] |
| Compatibility (-) > Relative Advantage (-) | *Logistical concerns were raised over the lack of menu space to display calories and the impact it would have on the aesthetics. Single outlet restaurant owners not displaying calories felt that it would “clutter” the menu and “bring down the standard” of the restaurant.* [Author interpreted summary (Geaney et al 2015 [[1](#_ENREF_1)])] | [[1](#_ENREF_1)] |
| Compatibility (-) > Adapting the Intervention (+) | *There were instances where variations occurred, for example, because different members of staff prepared products – even when using the same recipe cards. This left businesses with the dilemma of how to deal with these types of variation. Where this happened businesses tended to take a ‘common sense’ view, deciding on an average CI for the product.* [Author interpreted summary (Clegg et al 2009 [[10](#_ENREF_10)])] | [[10](#_ENREF_10)] |
| Compatibility (-) AND Design Quality & Packaging (-) > Adapting the Intervention (+) | *One of the barriers identified in the menu restriction theme (Table I) which was a genuine concern prior and post implementation, was that nutrition labelling did take up more space on the actual menu. However, staff became better at typographically presenting the information on the menu, ensuring it was at the point of purchase where it would be most effective.* [Author interpreted summary (Zick et al 2010 [[9](#_ENREF_9)])] | [[4](#_ENREF_4), [9](#_ENREF_9)] |
| Compatibility (-) > Scaling Up (+) | *Displaying CI only on selected items allowed businesses to introduce CI gradually, as they often felt that to cover all dishes would be too much at once. This was particularly the case for those with very large and/or frequently rotating menus.* [Author interpreted summary (Ray et al 2013 [[5](#_ENREF_5)])] | [[5](#_ENREF_5)] |
| Available Resources (-) > Relative Priority (-) | *Not a priority, time won’t allow it.* [Quote from foodservice business participant (Shupe 2013 [[12](#_ENREF_12)])] | [[12](#_ENREF_12)] |
| Available Resources (-) > Access to Knowledge & Information (-) | *One of the key challenges identified by participating food businesses in continuing the Caloriewise scheme once the pilot ended, and in rolling out the scheme to other premises, was that of obtaining calorie information for their products. Partly this was an issue of limited in-house technical expertise and partly an issue of the time/resources required to acquire the information.* [Author interpreted summary (Ray et al 2013 [[5](#_ENREF_5)])] | [[5](#_ENREF_5), [12](#_ENREF_12)] |
| Engaging: External Change Agent (-) > Access to Knowledge & Information (-) | *A respondent said that the information from the FSA [Food Standards Agency] was detailed but quite confusing for people who had never been involved in calorie labelling before*…[Author interpreted summary (Ray et al 2013 [[5](#_ENREF_5)])] | [[1](#_ENREF_1), [5](#_ENREF_5), [6](#_ENREF_6)] |
| Engaging: External Change Agent (+) > Access to Knowledge & Information (+) | *The Public Health Dietitians' knowledge and supportive role in the menu labelling pilot was found to be critical for enabling restaurant participation and engagement. Restaurants appreciated the flexibility, receptiveness, and technical knowledge of Public Health Dietitians throughout the pilot.* [Author interpreted summary (Toronto Public Health 2015 [[6](#_ENREF_6)])] | [[1](#_ENREF_1), [3-6](#_ENREF_3), [10](#_ENREF_10), [13](#_ENREF_13)] |
| Engaging: External Key Stakeholder (-) > Access to Knowledge & Information (-) | *Those displaying calories highlighted that discrepancy in ….. calorie information obtained from suppliers’ hindered implementation of accurate calorie information.* [Author interpreted summary (Geaney et al 2015 [[1](#_ENREF_1)])]  *Obtaining information from suppliers could take more time than business had anticipated, which in turn led to delays in providing calorie information in some outlets.* [Author interpreted summary (Clegg et al 2009 [[10](#_ENREF_10)])] | [[1](#_ENREF_1), [5](#_ENREF_5), [10-12](#_ENREF_10)] |
| Engaging: External Key Stakeholder (+) > Access to Knowledge & Information (+) | *… if the scheme were taken forward, a more structured approach to bringing suppliers on board would be essential: “It will need support from the suppliers in order to ensure you have as complete a database as possible … if rolling this out further, it would require a more targeted approach in terms of getting all the catering suppliers on board, providing the details of their products.* [Quote from foodservice business participant (Ray et al 2013 [[5](#_ENREF_5)])] | [[5](#_ENREF_5)] |
| Consumer Needs & Resources (-) > Tension for Change (-) | *My customers never request this information so I am not convinced it is needed.* [Quote from foodservice business participant (Ottawa Public Health 2013 [[3](#_ENREF_3)])] | [[3](#_ENREF_3)] |
| Consumer Needs & Resources (-) > Compatibility (-) | *In our business it is almost impossible to standardise a portion – often customers ask for extra potatoes or vegetables and we are happy to oblige – how can we standardise this?* [Quote from foodservice business participant (FSAI 2012 [[4](#_ENREF_4)])] | [[4](#_ENREF_4)] |
| Consumer Needs & Resources (-) > Goals & Feedback (-) > Relative Advantage (-) | *May even result in loss of business as a result of a negative dining experience.* [Author interpreted summary (Geaney et al 2015 [[1](#_ENREF_1)])] | [[1](#_ENREF_1)] |
| Consumer Needs & Resources (+) > Relative Advantage (+) | *For one business, taking part in the scheme was viewed as a potential way of improving their customer base by attracting more female customers; the business believed that CI would be of more interest to women than to men.* [Author interpreted summary (FSAI 2012 [[4](#_ENREF_4)])].  *By declaring calories, you’re being open and straight up with the customer; they will have more confidence in you.....they will have more confidence going to your company rather than someone else’s company.* [Quote from managing director at fast food chain (Geaney et al 2015 [[1](#_ENREF_1)])] | [[1](#_ENREF_1), [4](#_ENREF_4)] |
| Consumer Needs & Resources (-) AND Design Quality & Packaging (-) > Adapting the Intervention (+) | *Companies wanted the CI to be user-friendly for their customers, therefore, they wanted their displays to give enough information, but not too much so that people were overwhelmed (this led to some companies deciding not to display CI for products where there were many options, such as coffees).* [Author interpreted summary (Clegg et al 2009 [[10](#_ENREF_10)])] | [[1](#_ENREF_1), [10](#_ENREF_10)] |
| Economic Climate (-) > Relative Advantage (-) AND Relative Priority (-) | *The current economic climate was also felt to be an additional disincentive to participation: “… at this time in the financial cycle, I’m sure a lot of restaurants out there will go … ‘We need bums on seats and is the Caloriewise scheme going to work, is that going to do it for us?’ So that’s the hard-nosed financial reality in restaurants at the moment.’* [Author interpreted summary and quote from restaurant head office (Ray et al 2013 [[5](#_ENREF_5)])] | [[5](#_ENREF_5)] |
| External Policy & Incentives (-) > Adapting the Intervention (+) | *Worries were still expressed by companies that if Trading Standards, or a pressure group took a meal away for analysis it might vary from the CI displayed, and that action could be taken against them. One business had put a caveat on their menus stating that as food was prepared from fresh ingredients on the premises there might be some variation from the calorific values stated.* (Clegg et al 2009 [[10](#_ENREF_10)])] | [[10](#_ENREF_10)] |
| **Symbols: > = leading to, (-) = barrier, (+) = facilitator** | | |

**References**

1. Geaney F, Kelly C, Scotto Di Marrazzo J, Gilgan L, McCarthy M, Perry IJ. Evaluation of the uptake of voluntary calorie posting on menus in Ireland. Dublin: Department of Health, 2015.

2. Mah CL, Vanderlinden L, Mamatis D, Ansara DL, Levy J, Swimmer L. Ready for policy? Stakeholder attitudes toward menu labelling in Toronto, Canada. Can J Public Health. 2013;104:e229-34.

3. Ottawa Public Health. Report on Ottawa Restaurant Survey. Ottawa: Ottawa Public Health; 2013.

4. Food Safety Authority of Ireland (FSAI). Calories on menus in Ireland. A report on a national consultation. Dublin: FSAI; 2012.

5. Ray K, Clegg S, Davidson R, Vegeris S. Evaluation of Caloriewise: A Northern Ireland pilot of the display of calorie information in food catering businesses. Northern Ireland: Food Standards Agency, 2013.

6. Toronto Public Health. Voluntary Menu Labelling Pilot Project: Final Report. Ontario, Canada: Toronto Public Health; 2015.

7. Maestro V, Salay E. Restaurant nutrition and health information in the municipality of Campinas, São Paulo, Brazil: expectations of managers with respect to benefits and obstacles. Journal of Foodservice. 2008;19:262-9.

8. Din N, Zahari MSM, Othman CN, Abas R. Restaurant operator's receptiveness towards providing nutritional information on menu. Procedia Soc Behav Sci 2012;50:699-709.

9. Zick A, Wake Y, Reeves S. Nutrition labelling in restaurants: a UK-based case study. NUFS. 2010;40:557-65.

10. Clegg S, Jordan E, Slade Z. An Evaluation of Provision of Calorie Information by Catering Outlets. United Kingdom: Food Standards Agency, 2009.

11. Almanza BA, Nelson D, Chai S. Obstacles to nutrition labeling in restaurants. J Am Diet Assoc. 1997;97:157-61.

12. Shupe E. Obstacles to Participation in Menu Labeling Observed by the Independent Foodservice Establishments [Degree of Doctor of Philosophy]. Minnesota, United States: Walden University; 2013.

13. Britt JW, Frandsen K, Leng K, Evans D, Pulos E. Feasibility of voluntary menu labeling among locally owned restaurants. Health Promot Pract. 2011;12:18-24.

14. Jeong JY, Kim E, Yang IS, Ham S. Motivators and Barriers to Provision of Nutritional Information in Restaurants. Korean Journal of Hospitality & Tourism 2015;24:227-43.
