# Supplementary material for: Barriers and facilitators to implementation of menu labelling interventions from a food service industry perspective: a mixed methods systematic review
Source: Int J Behav Nutr Phys Act. 2020 Apr 15;17:48. doi: 10.1186/s12966-020-00948-1 (PMC7161210; doi:10.1186/s12966-020-00948-1)
Supplement: Supplementary file 9 — Additional file 9. Sensitivity analysis results for each construct. This file provides results of the sensitivity analysis. [file 12966_2020_948_MOESM9_ESM.docx]

| **Additional file 9** Sensitivity analysis results for each construct | | | | | |
| --- | --- | --- | --- | --- | --- |
| **CFIR Domain** | **CFIR Construct** | **CFIR sub-construct** | **Study Methodology** | **Study Quality** | **Study Location** |
| Intervention Characteristics | Intervention source |  | Qual & Mix Methods only | 50% & 75% only | Canada, Ireland & UK only |
|  | Evidence strength and quality |  | All methodology | 25%, 50% & 75% only | Canada, Ireland & UK only |
|  | Relative advantage |  | All methodology | All study quality | All locations |
|  | Complexity |  | All methodology | 25%, 50% & 75% only | USA, Canada, Ireland & UK only |
|  | Design quality and packaging |  | All methodology | All study quality | USA, Ireland & UK only |
|  | Cost |  | All methodology | 25%, 50% & 75% only | USA, Canada, Ireland, UK, Brazil, Malaysia, South Korea only |
| Outer Setting | Consumer needs and resources |  | All methodology | All study quality | All locations |
|  | Cosmopolitanism |  | Mix Methods only | 50% only | Ireland only |
|  | Peer pressure |  | All methodology | 0%, 50% & 75% only | USA, Canada, Ireland, UK, Netherlands & South Korea only. |
|  | External policy and incentives |  | All methodology | All study quality | All locations |
|  | Media & societal pressure*  Economic climate*  Educational system* |  | Qual & Quant only  Qual & Quant only  Qual & Mix Methods only | 0% & 50% only  25%, 50% & 75% only  50% & 75% only | USA, Ireland & South Korea only  Canada, Ireland, UK only  Ireland & UK only |
| Inner Setting | Structural characteristics |  | All methodology | All study quality | Canada, Ireland, UK, Netherlands & Brazil only |
|  | Networks and communications |  | Qual only | 50% only | UK only |
|  | Implementation climate |  |  |  |  |
|  |  | Tension for change | All methodology | 25% & 50% only | USA, Canada, Ireland, South Korea & Malaysia only |
|  |  | Compatibility | All methodology | 25%, 50% & 75% only | USA, Canada, Ireland, UK, South Korea & Brazil only |
|  |  | Relative priority | All methodology | 50% & 75% only | USA, Canada, Ireland & UK only |
|  |  | Incentives and rewards | Quant only | 50% only | Canada only |
|  |  | Goals and feedback | All methodology | All study quality | USA, Canada, Ireland, UK, South Korea & Malaysia only |
|  | Readiness for implementation |  |  |  |  |
|  |  | Leadership support | All methodology | 0, 50 & 75% only | Canada, Ireland, UK and Netherlands only |
|  |  | Available resources | All methodology | 25, 50 & 75% only | USA, Canada, Ireland, UK, Malaysia & South Korea only |
|  |  | Access to knowledge and information | All methodology | All study quality | USA, Canada, Ireland, UK, Netherlands, Malaysia & Brazil only |
| Characteristics of Individuals | Knowledge and beliefs |  | All methodology | All study quality | USA, Canada, Ireland & UK only |
|  | Other personal attributes |  | Qual & Quant only | 50% only | USA & Canada only |
| Process | Engaging |  | Qual only | 75% only | UK only |
|  |  | Opinion leaders | Qual Only | 75% only | UK only |
|  |  | External change agents | All methodology | All study quality | USA, Canada, Ireland & UK only |
|  |  | Internal key stakeholders* | All methodology | 50% & 75% only | USA, Canada, Ireland & UK only |
|  |  | External key stakeholders* | All methodology | 25%, 50% & 75% only | USA, Canada, Ireland & UK only |
|  | Executing |  | Quan & Mix Methods only | 50% only | Canada, Ireland & South Korea only |
|  | Reflecting & Evaluating |  | Quant only | 50% only | UK & Netherlands only |
|  | Adapting the Organisation* |  | Qual & Mixed Methods only | 50% & 75% only | Ireland & UK only |
|  | Adapting the Intervention* |  | All methodology | All study quality | USA, Canada, Ireland, UK & Malaysia only |
|  | Trialing* |  | Qual only | 75% only | UK only |
|  | Scaling up* |  | Qual only | 75% only | UK only |

*New construct developed following inductive analysis. Qual = qualitative, Quant = quantitative, Mix Methods = mixed methods. All study quality = 0%, 25%, 50% and 75% quality rating scores.
